# Imbalance of flight–freeze responses and their cellular correlates in the *Nlgn3*^*−/y*^ rat model of autism

**DOI:** 10.1186/s13229-022-00511-8

**Published:** 2022-07-18

**Authors:** Natasha J. Anstey, Vijayakumar Kapgal, Shashank Tiwari, Thomas C. Watson, Anna K. H. Toft, Owen R. Dando, Felicity H. Inkpen, Paul S. Baxter, Zrinko Kozić, Adam D. Jackson, Xin He, Mohammad Sarfaraz Nawaz, Aiman Kayenaat, Aditi Bhattacharya, David J. A. Wyllie, Sumantra Chattarji, Emma R. Wood, Oliver Hardt, Peter C. Kind

**Affiliations:** 1 Centre for Discovery Brain Sciences, Simons Initiative for the Developing Brain, University of Edinburgh, Hugh Robson Building, 5 George Square, Edinburgh, EH8 9XD UK; 2 Centre for Brain Development and Repair, InStem, National Centre for Biological Sciences, Bangalore, Karnataka 560065 India; 3 Dementia Research Institute, University of Edinburgh, Edinburgh, EH8 9XD UK; 4 Department of Psychology, McGill University, Montréal, QC H3A 1B1 Canada; 5 The University of Transdisciplinary Health Sciences and Technology, Bangalore, Karnataka 560065 India

**Keywords:** Fear, Freezing, Flight, Autism, Intellectual disability, Periaqueductal grey, Neuroligin-3

## Abstract

**Background:**

Mutations in the postsynaptic transmembrane protein neuroligin-3 are highly correlative with autism spectrum disorders (ASDs) and intellectual disabilities (IDs). Fear learning is well studied in models of these disorders, however differences in fear response behaviours are often overlooked. We aim to examine fear behaviour and its cellular underpinnings in a rat model of ASD/ID lacking *Nlgn3*.

**Methods:**

This study uses a range of behavioural tests to understand differences in fear response behaviour in *Nlgn3*^−/y^ rats. Following this, we examined the physiological underpinnings of this in neurons of the periaqueductal grey (PAG), a midbrain area involved in flight-or-freeze responses. We used whole-cell patch-clamp recordings from ex vivo PAG slices, in addition to in vivo local-field potential recordings and electrical stimulation of the PAG in wildtype and *Nlgn3*^−/y^ rats. We analysed behavioural data with two- and three-way ANOVAS and electrophysiological data with generalised linear mixed modelling (GLMM).

**Results:**

We observed that, unlike the wildtype, *Nlgn3*^−/y^ rats are more likely to response with flight rather than freezing in threatening situations. Electrophysiological findings were in agreement with these behavioural outcomes. We found in ex vivo slices from *Nlgn3*^−/y^ rats that neurons in dorsal PAG (dPAG) showed intrinsic hyperexcitability compared to wildtype. Similarly, stimulating dPAG in vivo revealed that lower magnitudes sufficed to evoke flight behaviour in *Nlgn3*^−/y^ than wildtype rats, indicating the functional impact of the increased cellular excitability.

**Limitations:**

Our findings do not examine what specific cell type in the PAG is likely responsible for these phenotypes. Furthermore, we have focussed on phenotypes in young adult animals, whilst the human condition associated with *NLGN3* mutations appears during the first few years of life.

**Conclusions:**

We describe altered fear responses in *Nlgn3*^*−/y*^ rats and provide evidence that this is the result of a circuit bias that predisposes flight over freeze responses. Additionally, we demonstrate the first link between PAG dysfunction and ASD/ID. This study provides new insight into potential pathophysiologies leading to anxiety disorders and changes to fear responses in individuals with ASD.

**Supplementary Information:**

The online version contains supplementary material available at 10.1186/s13229-022-00511-8.

## Introduction

Autism spectrum disorders (ASDs) and intellectual disabilities (IDs) are a complex, heterogeneous group of disorders that are poorly understood in terms of their underlying cellular and circuit pathophysiology. Single-gene mutations account for a large proportion of cases where individuals present with ASD and co-occurring moderate to severe ID [16, 17, 61] and of these, mutations in synaptic proteins have been repeatedly implicated [84]. Mutations in the gene encoding the synaptic protein neuroligin-3 (NLGN3) were originally linked to ASD in 2003 [34], and point mutations in *NLGN3* have since been shown to be associated with ASD/ID in several studies [33, 37, 43, 46, 52, 54, 64, 66, 73, 78, 79, 81–84]. The majority of *NLGN3* mutations identified in humans result in complete or near-complete loss of the NLGN3 protein [13, 15, 37, 52, 54, 65, 66]. NLGN3 is a scaffolding protein expressed at both excitatory and inhibitory synapses where it plays a key role in synaptic development, function, and maintenance [10, 71]. Mouse models of both null and point mutations in *Nlgn3* lead to behavioural phenotypes as well as alteration synaptic function and plasticity, although the precise nature of these phenotypes differs in a mutation-specific manner [21, 65, 25, 50, 85]. These synaptic deficits have been shown to underlie circuit and behavioural dysfunction [6, 21, 32, 51, 58, 65]. More recently, an in vivo study in *Nlgn3* KO mice demonstrated an increase in excitability of neurons in CA2 linked to social cognition deficits [47], raising the intriguing possibility that mutations in *Nlgn3* could alter the intrinsic physiology of neurons.

Co-occurrence of anxiety and altered emotional responses in individuals with ASD ranges from rates of 11–84% depending on the severity of ASD [44, 63, 76]. Clinically, the presentation of anxiety disorders and phobias are so prevalent in individuals with ASD that they are considered an auxiliary feature of the autism spectrum, and often used as part of diagnosis [38]. However, relatively little is known about the role of NLGN3 in the circuits responsible for fear and emotional learning. Emotional responses have been modelled in animals using fear conditioning paradigms and rat models of ASD and ID have been reported to show reduced freezing behaviour during in fear learning or extinction [12, 30, 35, 40, 53]. Indeed, decreased freezing behaviour during fear conditioning has been shown in *Nlgn3*^*−/y*^ mice [53], but not in *Nlgn3* R451C mice [12, 35], and results from the rat model are unclear [30]. All these studies focus on freezing behaviour as the primary readout of fear learning. However, freezing behaviour is not the only fear response exhibited by rodents, or indeed in humans, and hence altered fear expression could be an equally plausible explanation for the reduced freezing. Fight–flight–freeze responses are relatively well characterised, and the decision of which of these responses manifests depends on the context of the fearful situation in which it occurs [26].

The importance of the periaqueductal grey (PAG) in regulating the execution of fear responses has been well demonstrated, both by seminal studies from the 1980s and 1990s [4, 5, 23, 24,, 60, 67, 86], and by more recent work investigating fear circuitry [2, 18, 22, 36, 41, 59, 56, 57, 74, 68, 75]. The PAG receives and integrates inputs from many brain regions, including the hypothalamus [74], amygdala [39, 68], medial-prefrontal cortex [59], and superior colliculus [22], resulting in the expression of flight-or-freeze fear responses. The role of the PAG in contributing to the pathophysiology of ASD has not yet been elucidated. This study demonstrates an imbalance in fear responses in *Nlgn3*^*−/y*^ rats and an alteration in cellular excitability in the PAG.

## Results

### Confirmation of the ***Nlgn3***^***−/y***^ rat model

**Fig. 1:**
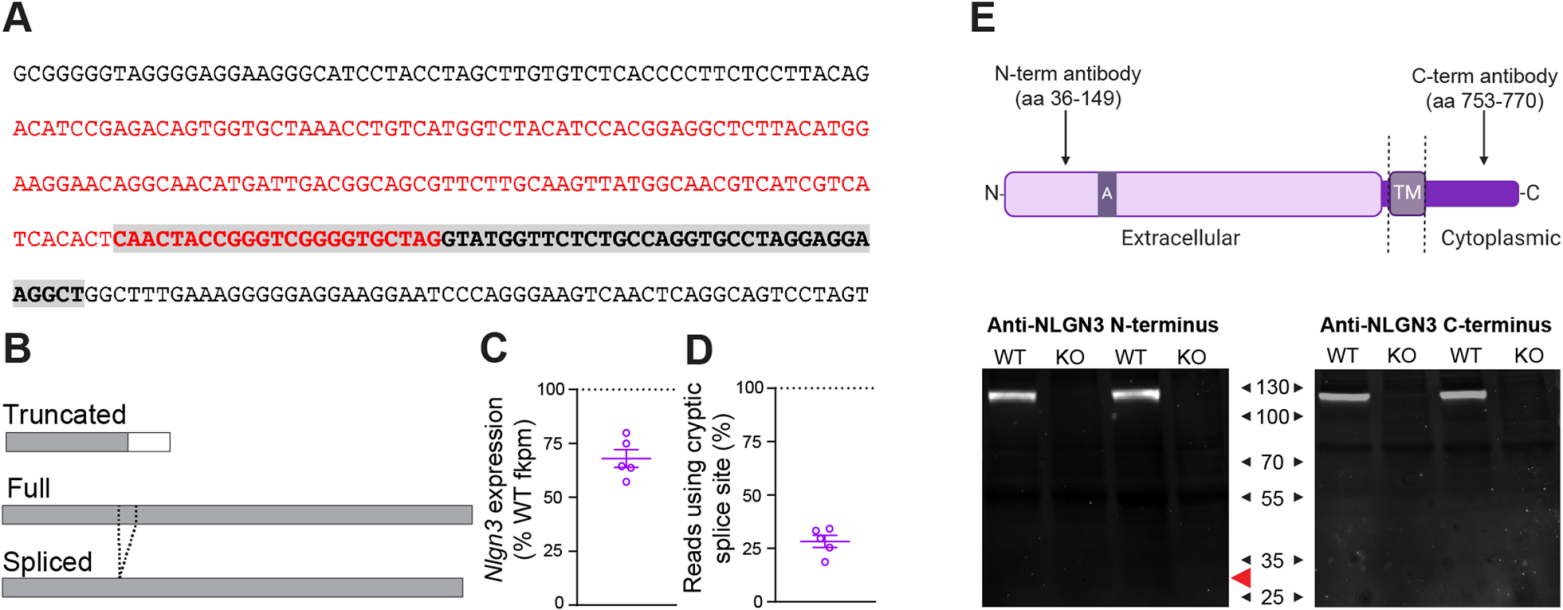
Confirmation of the *Nlgn3*^*−/y*^ rat model. **A** Base sequence of deletion in exon 5. Red text denotes exon, highlighted grey text denotes deletion location. **B** Schematic of potential truncated, full and spliced variants of the NLGN3 protein. Grey regions indicated amino acid sequence shared with the full-length isoform, dotted lines indicate a 17 amino acid section of the full-length *Nlgn3* that is missing in the spliced form. **C** *Nlgn3* mRNA expression in *Nlgn3*^*−/y*^ animals expressed as a percentage of WT (WT n = 6, KO n = 5). **D** Percentage of *Nlgn3*^*−/y*^ RNA-seq reads at the cryptic splice site which splice. (WT n = 6, KO n = 5). **E** Schematic illustrating antibody binding sites to WT NLGN3 protein. Western blot of cortical WT and *Nlgn3*^*−/y*^ tissue using anti-NLGN3 N-terminus and anti-NLGN3 C-terminus. No NLGN3 protein of any form was found in *Nlgn3*^*−/y*^ rats (WT n = 2, KO n = 2). Red arrow denotes expected band location if short isoform was present. No presence of the predicted short isoform was detected. Data represented as mean ± SEM, clear dots represent individual animals

A rat model of NLGN3 deficiency was created by zinc-finger nuclease targeting of exon 5 of *Nlgn3*, leading to a 58 bp deletion (Envigo [30], Fig. 1A). RNA sequencing revealed a ~ 25% loss of *Nlgn3* mRNA in *Nlgn3*^*−/y*^ rats vs WTs (Fig. 1C). It revealed the presence of two novel mRNA variants, a truncated “short” isoform, caused by transcription through the deletion site until a stop codon is reached in the adjacent intron, and a long isoform, caused by a cryptic splice site upstream of the targeted deletion, with ~ 25% of RNA-seq reads splicing at this locus to the next exon (Fig. 1D). The short isoform could encode a ~ 30kD protein, whereas the long isoform encodes a predicted protein product 17 amino acids shorter than the full-length protein (Fig. 1B). As both these abnormal potential *Nlgn3* isoforms are predicted to contain the N-terminus, but not the C-terminus, of NLGN3, we utilised Western blotting to probe for these (Fig. 1E, Additional file 1: Fig. S9(A–B). We found no presence of NLGN3 protein in *Nlgn3*^*−/y*^ cortical homogenate using either C-terminus or N-terminus-specific NLGN3 antibodies (Fig. 1E), indicating the novel mRNA variants are not translated to a protein product or generate a highly unstable protein.

### *Nlgn3*^*−/y*^ rats exhibit reduced classic freezing behaviour during conditioning and recall phases of auditory fear conditioning

**Fig. 2:**
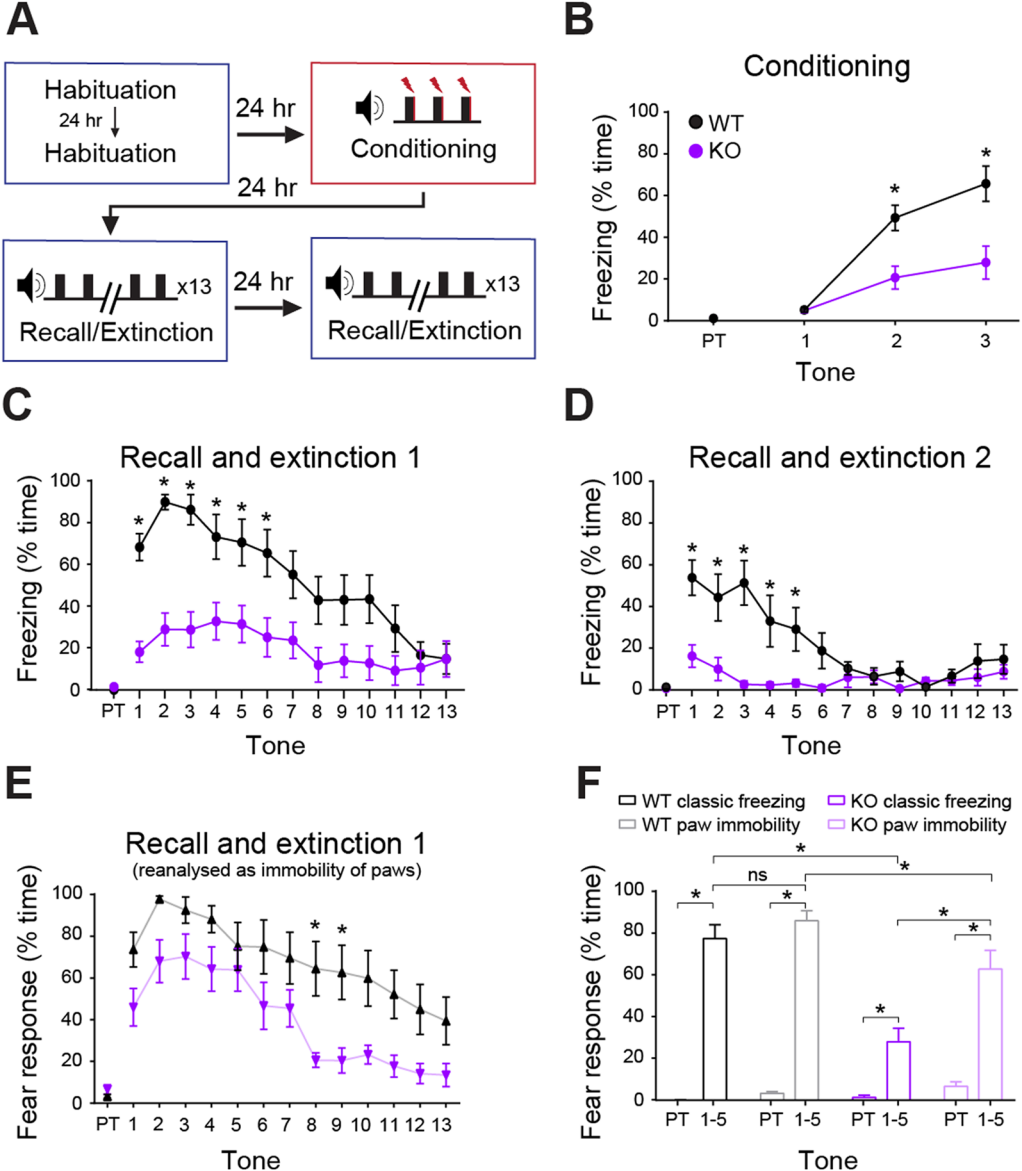
*Nlgn3*^*−/y*^ rats display reduced classic freezing behaviour in an auditory fear conditioning paradigm. **A** Schematic of the auditory fear conditioning protocol. **B** *Nlgn3*^*−/y*^ rats show less classic freezing behaviours during the conditioning phase (*p* < 0.0001, F_(1, 22)_ = 6.61, repeated measures two-way ANOVA, WT n = 12, KO n = 12). PT: Pre-tone. **C** *Nlgn3*^*−/y*^ rats show less classic freezing behaviours during the recall and extinction phase (p = 0.001, F_(1, 22)_ = 13.36, post hoc two-way ANOVA, WT n = 12, KO n = 12). **D** *Nlgn3*^*−/y*^ rats show reduced classic freezing behaviours during the second extinction phase (*p* = 0.0009, F_(1, 22)_ = 14.61, repeated measures two-way ANOVA, WT n = 12, KO n = 12). **E** When analysed as “immobility response” (all four paws unmoving but allowing for movement of head and neck) *Nlgn3*^*−/y*^ rats show significantly increased response to CS in comparison with classic freezing scoring (p = 0.004, F_(1, 22)_ = 13.31, post hoc two-way ANOVA, KO n = 12). WT rats also show significantly increased paw immobility response in comparison with classic freezing behaviour (*p* = 0.019, F_(1, 22)_ = 7.58, post hoc two-way ANOVA, WT n = 12). Expression of paw immobility response behaviour is significantly lower in *Nlgn3*^*−/y*^ rats in comparison with WT (p < 0.0001, F_(1, 22)_ = 3.26, post hoc two-way ANOVA, WT n = 12, KO n = 12). **F** Percentage time exhibiting a fear response (defined as either classic freezing (black, purple) or immobility of paws (grey, pink) for pre-tone and average of tones 1–5 of recall shows a significant interaction between genotype, method of scoring, and presence of CS (*p* = 0.012, F_(1, 22)_ = 7.52, three-way ANOVA, WT n = 12, KO n = 12). Both WT and *Nlgn3*^*−/y*^ rats display significant response to the CS (WT classic freezing: *p* < 0.0001, WT paw immobility: *p* < 0.0001, KO classic freezing: *p* = 0.008, KO paw immobility: *p* < 0.0001, post hoc Bonferroni-corrected paired t-tests). Scoring method does not affect fear response behaviour during recall for WT rats (p = 0.24, post hoc Bonferroni-corrected paired t-test) however a significantly higher paw immobility response is expressed by *Nlgn3*^*−/y*^ rats in comparison with classic freezing behaviour (*p* < 0.0001, post hoc Bonferroni-corrected paired t-test). Data represented as mean ± SEM

*Nlgn3*^*−/y*^ mice have been reported to show reduced freezing during both cued and contextual fear conditioning tasks [53]. We found that relative to WT controls, *Nlgn3*^*−/y*^ rats also exhibit reduced freezing behaviour during the conditioning (*p* < 0.0001, F_(1, 22)_ = 6.61), recall (*p* = 0.001, F_(1, 22)_ = 13.36), and extinction (*p* = 0.0009, F_(1, 22)_ = 14.61) phases of an auditory-cued fear conditioning task (Fig. 2A–D) and during a contextual fear conditioning task (Additional file 1: Fig. S1A–D), conditioning *p* = 0.025, F_(1, 25)_ = 5.67,recall *p* < 0.0001, F_(1, 25)_ = 26.61). Whilst *Nlgn3*^*−/y*^ rats did not exhibit high levels of freezing during recall, defined as no movement except for breathing, they did appear to respond to the tone by decreasing their overall movement. Therefore, we redefined fear response as immobility of the paws and torso but allowing for head movements. This reanalysis revealed that *Nlgn3*^*−/y*^ rats show a more similar fear learning and extinction profile to WTs, although still significantly reduced (Fig. 2E–F, *p* < 0.0001, F_(1, 22)_ = 3.23, Additional file 1: Fig. S1D, *p* < 0.0001, F_(1, 25)_ = 20.65, Additional file 1: Fig. S2A, *p* = 0.008, F _(1, 22)_ = 8.333). Examination of freezing and paw immobility during the first 5 CS presentations shows significantly reduced recall in *Nlgn3*^*−/y*^ rats relative to WTs (Fig. 2F, *p* = 0.012, F_(1, 22)_ = 7.52). However, *Nlgn3*^*−/y*^ rats display a significantly higher response to the CS when considering immobility of paws only in comparison with classic freezing (Fig. 2F, *p* < 0.0001). This effect was not seen in WT animals (Fig. 2F, *p* = 0.24). As hyperactivity could be a confounding the interpretation of these data, we tested locomotion in the same cohort of WT and *Nlgn3*^*−/y*^ rats in an open field arena before running the fear conditioning paradigm. Analysis of open field behaviour revealed no differences in movement between WT and *Nlgn3*^*−/y*^ rats on any of the four days tested (Additional file 1: Fig. S3A, *p* = 0.29, F_(1, 22)_ = 1.19. Furthermore, on a marble interaction task, time interacting with marbles was not different between WT and *Nlgn3*^*−/y*^ rats (Additional file 1: Fig. S3E, *p* = 0.09). These findings indicate that *Nlgn3*^*−/y*^ rats, despite showing reduced freezing behaviour, still form the association between tone and shock but may be expressing their fear in a different manner.

### *Nlgn3*^*−/y*^ rats show improved learning of the shock-zone in the active place avoidance task

**Fig. 3:**
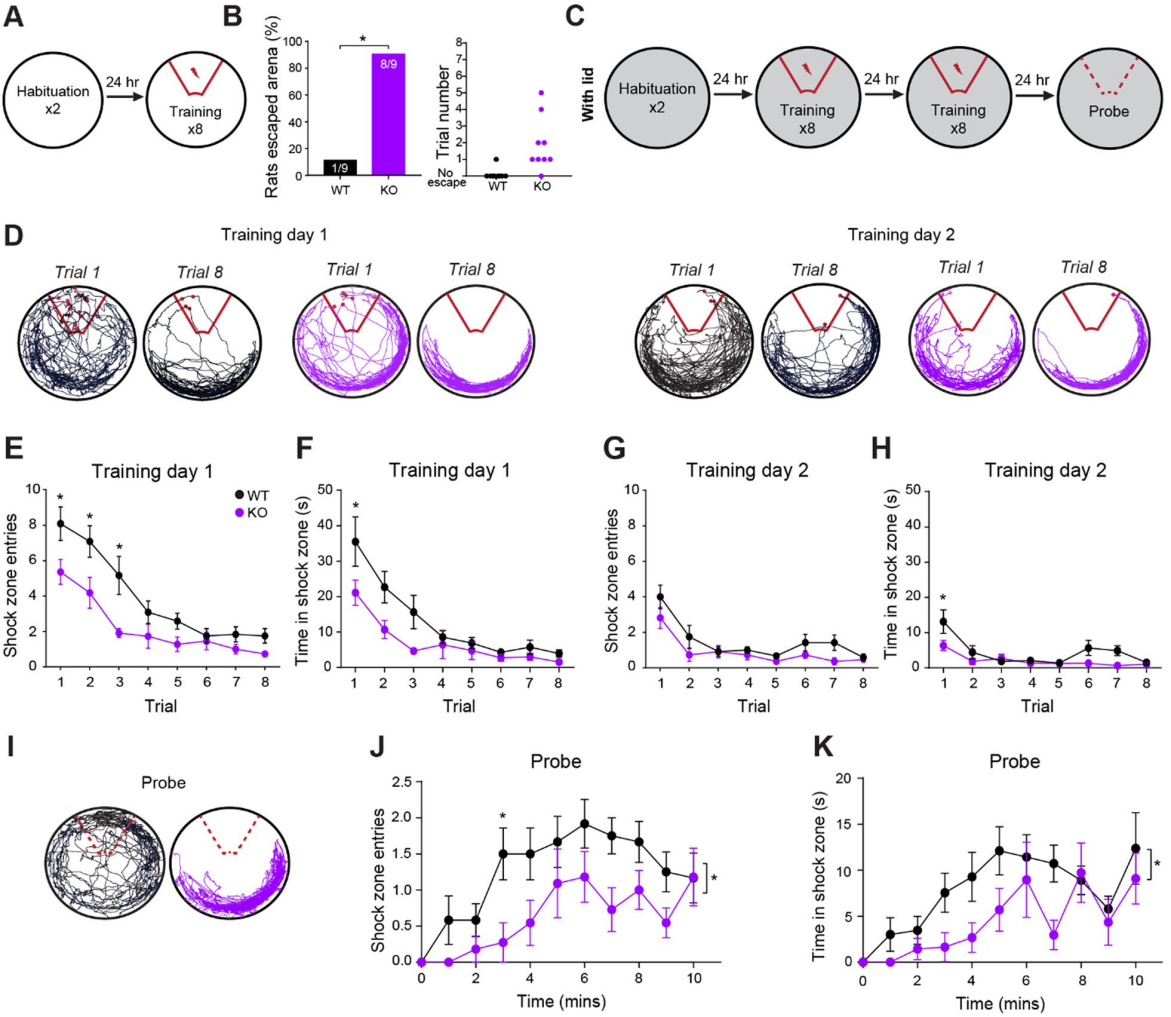
*Nlgn3*^*−/y*^ rats show faster learning and prolonged avoidance of the shock-zone in an active place avoidance task. **A** Schematic depicting habituation day and first training session of active place avoidance task (no lid present on arena). **B** 88.9% *Nlgn3*^*−/y*^ and 11.1% WT rats jumped out of the arena following 0.2 mA foot-shocks given over the 8 training trials training (*p* = 0.0034, Fisher’s exact test, WT n = 9, KO n = 9). Training trial number on which each rat escaped is displayed on right. **C** Schematic of the active place avoidance task, with added lid. **D** Representative track  plots for WT and* Nlgn3*^-/y^ rats in trials 1 and 8 of training sessions 1 and 2.** E**,** F** *Nlgn3*^-/y^ rats enter the shock-zone significantly fewer times during training session 1 (*p* = 0.0045, F_(1, 21)_ = 10.09, repeated measures two-way ANOVA, WT n = 12, KO n = 11), and spend significantly less time in the shock-zone (*p* = 0.027, F_(1, 21)_ = 5.68 repeated measures two-way ANOVA, WT n = 12, KO n = 11).** G**,** H** *Nlgn3*^-/y^ rats enter the shock-zone significantly fewer times during training session 2 (*p* = 0.044, F_(1, 21)_ = 4.60, repeated measures two-way ANOVA, WT n = 12, KO n = 11), and spend significantly less time in the shock-zone (*p* = 0.025, F_(1, 21)_ = 5.80, repeated measures two-way ANOVA, WT n = 12, KO n = 11).** I** Representative track plots for WT and *Nlgn3*^*−/y*^ rats in the probe trial.** J**,** K** *Nlgn3*^-/y^ rats enter the shock-zone significantly fewer times during the probe trial (*p* = 0.0039, F_(1, 21)_ = 10.51, repeated measures two-way ANOVA, WT n = 12, KO n = 11), and spend significantly less time in the shock-zone (*p* = 0.045, F_(1, 21)_ = 4.53, repeated measures two-way ANOVA, WT n = 12, KO n = 11). Data represented as mean ± SEM

To further explore a potential role for NLGN3 in fear learning, we employed the active place avoidance (APA) task (Fig. 3A, C). During training, response to the low-ampere foot-shock differed between genotypes. Over the course of the 8 trials, 8/9 *Nlgn3*^*−/y*^ rats responding by jumping and escaping the arena altogether. Only 1/9 WT rats showed this behaviour (Fig. 3B, *p* = 0.0034). Once an animal escaped the arena the trial had to be ended as it was not possible to measure the time to learn the location of the shock-zone.

When testing was repeated in a modified arena with a lid to discourage jumping/escape behaviour, naïve cohorts of both WT and *Nlgn3*^*−/y*^ rats displayed no escape behaviour in response to the foot-shock, in addition to learning the location of the shock-zone by the end of the training sessions and could avoid it by actively remaining in the safe zone (Fig. 3D–H). *Nlgn3*^*−/y*^ rats displayed enhanced performance in this task; throughout training sessions 1 (TS1) and 2 (TS2) *Nlgn3*^*−/y*^ rats entered into the shock-zone significantly fewer times across trials (Fig. 3E, G, TS1: *p* = 0.0045, F_(1, 21)_ = 10.09, TS2: *p* = 0.044, F_(1, 21)_ = 4.60), and spent significantly less time in this zone (Fig. 3F, TS1: *p* = 0.027, F_(1, 21)_ = 5.68, TS2: *p* = 0.025, F_(1, 21)_ = 5.80) in comparison with WT rats. During habituation to the arena, distance travelled changed over habituation days (Additional file 1: Fig. S3C–D, *p* = 0.008, F_(3, 42)_ = 0.53), however *Nlgn3*^*−/y*^ rats showed no hyperactivity in comparison with WT (Additional file 1: Fig. S3C–D, Trial 1: WT vs *Nlgn3*^*−/y*^*,* p = 0.99, Trial 2: WT vs *Nlgn3*^*−/y*^*,* p = 0.90). Furthermore, during training in the presence of foot-shocks the locomotion was not different between WT and *Nlgn3*^*−/y*^ rats (Additional file 1: Fig. S3D, *p* = 0.59, F_(1, 21)_ = 0.29). During the probe trial, *Nlgn3*^*−/y*^ rats displayed significantly prolonged avoidance of previous shock-zone relative to WT animals, despite no shock being applied. *Nlgn3*^*−/y*^ rats entered the previous shock-zone fewer times on average (Fig. 3J, *p* = 0.0039, F_(1, 21)_ = 10.51), and spent less total time in this zone (Fig. 3K, *p* = 0.045, F_(1, 21)_ = 4.53) in comparison with WTs.

The ability of the *Nlgn3*^*−/y*^ rats to successfully learn the location of the shock-zone indicates that spatial memory is unaffected by the loss of NLGN3. However, the exaggerated escape behaviour of *Nlgn3*^*−/y*^ rats seen in the unmodified arena, along with the increased avoidance during the probe trial, suggests NLGN3 loss results in altered fear expression to the shock.

### *Nlgn3*^*−/y*^ rats display increased jumping behaviour during a shock-ramp test

**Fig. 4:**
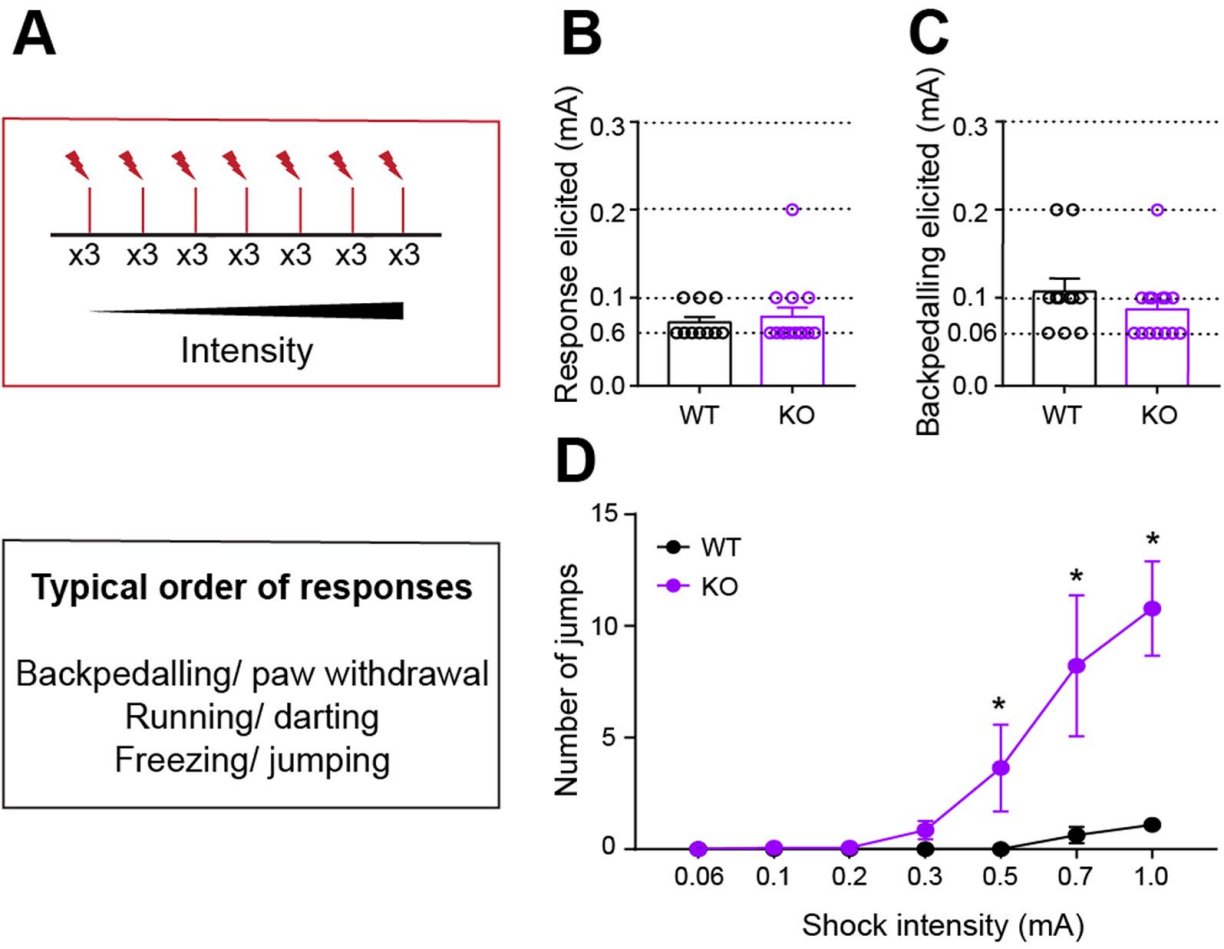
*Nlgn3*^*−/y*^ rats display increased jumping behaviour in response to electrical shocks. **A** Schematic of the shock-ramp test protocol and typical order of responses seen. **B** Lowest shock amplitude required to elicit a response of any kind was not different between WT and *Nlgn3*^*−/y*^ rats (p = 0.13, unpaired t-test, WT n = 11, KO n = 14). **C** Shock amplitude required to elicit backpedalling response was not different between WT and *Nlgn3*^*−/y*^ rats (p = 0.26, unpaired t-test, WT n = 11, KO n = 14). **D** *Nlgn3*^*−/y*^ rats display significantly more jumps in response to increasing intensity electrical foot-shocks (*p* = 0.0081, F_(1, 23)_ = 8.39, repeated measures two-way ANOVA, WT n = 11, KO n = 14). Data represented as mean ± SEM, clear dots represent individual animals

One possible explanation for the data described thus far is *Nlgn3*^*−/y*^ rats are hypersensitive to electrical shocks, and this difference in sensitivity leads to atypical fear response behaviour. To test this, we examined the response of naïve WT and *Nlgn3*^*−/y*^ rats to increasing intensities of foot-shocks (0.06 to 1 mA). Backpedalling and paw withdrawal were the most common initial behaviours observed when an animal first responded to a foot-shock (Fig. 4A). The minimum shock required to elicit any response, or to elicit a backpedalling response, was not different between *Nlgn3*^*−/y*^ and WT rats (Fig. 4B, *p* = 0.13, Fig. 4C, *p* = 0.26). This indicates *Nlgn3*^*−/y*^ rats are not hypersensitive to foot-shocks. Additionally, thermal stimulus-induced tail-flick response in *Nlgn3*^*−/y*^ rats was increased compared to WT rats (Additional file 1: Fig. S4B, *p* = 0.036), suggesting a decreased sensitivity to thermal pain in *Nlgn3*^*−/y*^ rats.

However, *Nlgn3*^*−/y*^ rats exhibited significantly more jumping behaviour than WT rats in response to the higher amplitude shocks (Fig. 4D, *p* = 0.0081, F_(1, 23)_ = 8.39), suggesting that *Nlgn3*^*−/y*^ rats tend to exhibit flight behaviour in response to foot-shocks. The greater incidence of jumping behaviour here in comparison with the fear conditioning paradigm shown in Fig. 1 is likely due to the repetitive, increasing nature of the foot-shocks given in the paradigm here. At the end of the ramp phase, the shock amplitude was reduced to assess sensitivity changes of the animals induced by the paradigm. Number of jumps to this lower shock intensity was not significantly different between WT and *Nlgn3*^*−/y*^ animals (Additional file 1: Fig. S4A, *p* = 0.35). These data further suggest that the loss of NLGN3 leads to increased flight behaviour in response to fearful stimuli.

### Dorsal, but not ventral, periaqueductal grey cells in *Nlgn3*.^*−/y*^ rats are intrinsically hyperexcitable ex vivo

We hypothesised that the increase in shock-elicited flight response in *Nlgn3*^*−/y*^ rats is due to altered physiological properties in the periaqueductal grey (PAG), a midbrain region previously shown to control fear expression [2, 18, 22, 36, 39, 41, 56, 57, 68, 75].

**Fig. 5:**
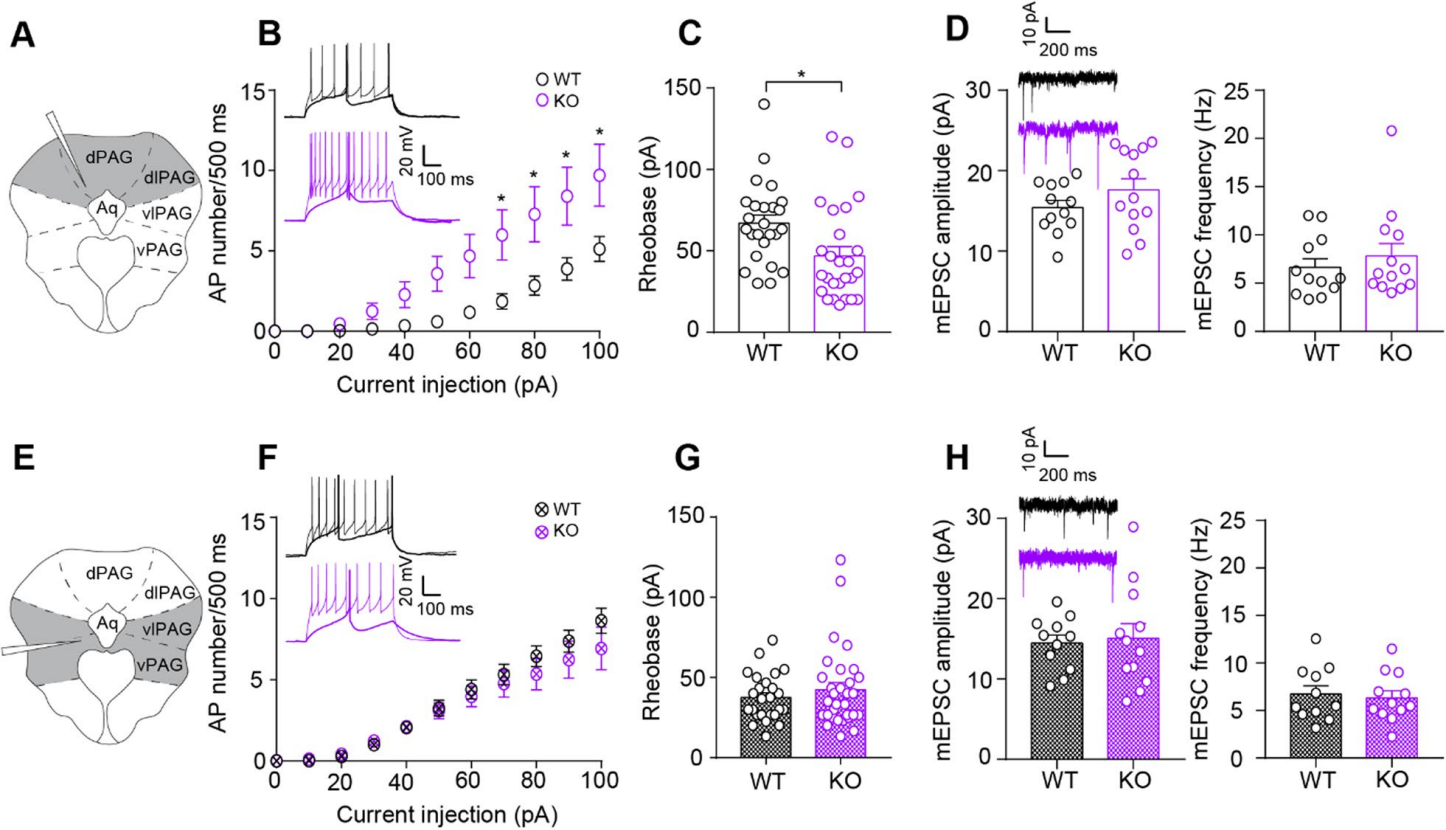
Hyperexcitability of dorsal, but not ventral PAG neurons in *Nlgn3*^*−/y*^ rats. **A**, **E** Schematics of PAG slice indicating area recorded from in grey. **B** dPAG cells from *Nlgn3*^*−/y*^ rats fire increased numbers of action potentials in response to increasing current injection steps (*p *= 0.018, F_(1, 17)_ = 6.87, repeated measures two-way ANOVA, WT n = 25 cells/ 10 rats, KO n = 26 cells/ 9 rats). Representative traces of rheobase and + 100 pA steps for WT (black) and *Nlgn3*^*−/y*^ (purple) dPAG cells. **C** dPAG cells from *Nlgn3*^*−/y*^ rats have lower rheobase potential than WT (*p* = 0.014, GLMM, WT n = 25 cells/ 10 rats, KO n = 26 cells/ 9 rats). **D** No change in mEPSC amplitude or frequency of dPAG neurons in *Nlgn3*^*−/y*^ rats compared to WT (amplitude: *p* = 0.28, frequency *p*= 0.61, GLMM, WT 12 cells/ 6 rats, KO 13 cells/ 6 rats). Representative traces of mEPSCs of dPAG cells from WT (black) and *Nlgn3*^*−/y*^ (purple) rats. **F** vPAG cells from *Nlgn3*^*−/y*^ and WT rats fire comparable numbers of action potentials in response to increasing current injection steps (*p* = 0.54, F_(1, 17)_ = 0.38, repeated measures two-way ANOVA, WT n = 24 cells/ 9 rats, KO n = 28 cells/ 10 rats). Representative traces of rheobase and + 100 pA steps for WT (black) and *Nlgn3*^*−/y*^ (purple) vPAG cells. **G** vPAG cells from *Nlgn3*^*−/y*^rats have a comparable rheobase potential to WT cells (*p* = 0.4, GLMM, WT n = 24 cells/ 9 rats, KO 28 cells/ 10 rats). **H** No change in mEPSC amplitude or frequency of vPAG neurons in *Nlgn3*^*−/y*^ rats in comparison to WT (amplitude: *p *= 0.78, frequency: *p* = 0.88, GLMM, WT n=24 cells/ 9 rats KO n=28 cells/ 10 rats). Data represented as mean ± SEM, dots represent individual cells

Using whole-cell patch-clamp recordings from acute slices, we found that cells in the dPAG fired an increased number of action potentials to incremental depolarising current injections in *Nlgn3*^*−/y*^ rats compared to WTs (Fig. 5B, *p* = 0.018, F_(1, 17)_ = 6.87). Rheobase current was also significantly decreased in *Nlgn3*^*−/y*^ cells (Fig. 5C, *p* = 0.014). No changes in the passive membrane properties or action potential threshold (Additional file 1: Fig. S5) were observed in *Nlgn3*^*−/y*^ rats, however the fast-afterhyperpolarisation potential was significantly decreased (Additional file 1: Fig. S5H, *p* = 0.0047). Conversely, vPAG cells recorded from *Nlgn3*^*−/y*^ and WT rats fired an equivalent number of action potentials (Fig. 5F, *p* = 0.54, F_(1, 17)_ = 0.38) and had an average rheobase current comparable to that of WT rats (Fig. 5G, *p* = 0.40). *Nlgn3*^*−/y*^ vPAG neurons did, however, display increased membrane time constants (Additional file 1: Fig. S5, *p* = 0.0095). In order to achieve optimal slice health, these recordings were performed in slices from rats ages 4–6 weeks, however a small dataset was also recorded in slices from animals ages 8–10 weeks in order to confirm the hyperexcitability in the dPAG perseveres into young adult *Nlgn3*^*−/y*^ rats. Indeed, we observed an increase in excitability of neurons in the dPAG of *Nlgn3*^*−/y*^ rats in comparison with WT (Additional file 1: Fig. S6A, *p* = 0.0094, F_(1, 9)_ = 10.82), but not in vPAG neurons (Additional file 1: Fig. S6B, *p* = 0.92, F_(1, 13)_ = 0.0097). This observed hyperexcitability of dPAG cells in *Nlgn3*^*−/y*^ rats may explain the increased flight and decreased freezing behaviour seen in these rats.

In addition to intrinsic excitability, the excitability of a neuron depends on the synaptic input it receives. We measured mEPSC amplitude and frequencies in dorsal and ventral PAG cells using whole-cell patch-clamp recordings in acute slices from *Nlgn3*^*−/y*^ and WT rats. We found that cells recorded from *Nlgn3*^*−/y*^ and WT rats had comparable mEPSC amplitudes and frequencies in both dPAG (Fig. 5D, amplitude: *p* = 0.28, frequency: *p* = 0.61) and vPAG (Fig. 5H, amplitude: *p* = 0.78, frequency: *p* = 0.88). Together, these data suggest that dPAG cells are intrinsically hyperexcitable, but do not appear to receive altered excitatory synaptic input.

### *Nlgn3*^*−/y*^ rats display normal tone-evoked LFP amplitudes in the PAG during fear recall

**Fig. 6:**
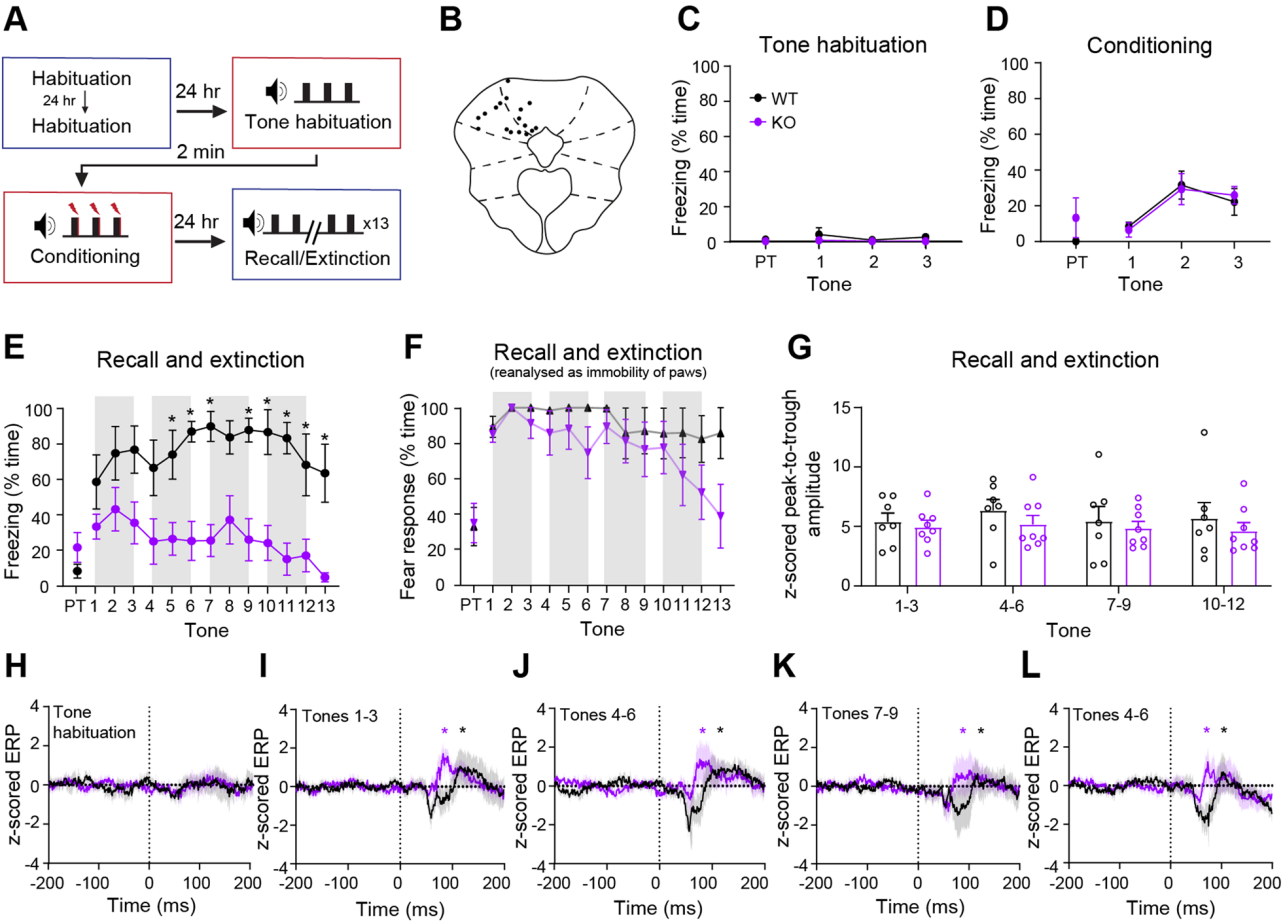
*Nlgn3*^*−/y*^ rats show similar dPAG ERP amplitudes to WT rats during an auditory fear conditioning paradigm. **A** Schematic of auditory fear conditioning paradigm including tone habituation session. **B** Approximate locations of recording sites in the PAG. Black dots represent lesion site after electrode removal for an individual animal. **C** Almost no classic freezing behaviour was seen during the tone habituation session (*p *= 0.13, F_(1, 13) _= 2.63, repeated measures two-way ANOVA, WT n = 7, KO n = 8). PT: Pre-tone. **D** WT and *Nlgn3*^*−/y*^ rats display similar levels of freezing behaviour during auditory fear conditioning (*p* = 0.54, F_(1, 13) _= 0.74, repeated measures two-way ANOVA, WT n = 7, KO n = 8). **E** *Nlgn3*^-/y^ rats display significantly lower freezing behaviour during fear recall in comparison with WT cage-mates (*p* = 0.032, F_(1, 13) _= 1.96, three-way ANOVA, WT n = 7, KO n = 8). Grey boxes represent tone-responses that have been averaged for panels (**H**–**K**).**F** When analysed as “immobility response” (all four paws unmoving but allowing for movement of head and neck) *Nlgn3*^*-/y*^ rats show no difference in this behaviour during fear recall (*p* = 0.001, F_(1, 7) _= 29.75, three-way ANOVA, KO n = 8) in comparison with WT controls (*p* = 0.12, F_(1, 6) _= 3.39, three-way ANOVA, WT n = 7). (*p* = 0.014, F_(1, 13) _= 8.036 (analysis method x genotype), *p* = 0.28, F_(12, 156) _= 1.21 (tone x analysis method), *p *= 0.093, F_(12, 156) _= 1.62 (tone x genotype), three-way ANOVA, WT n = 7, KO n = 8). **G** No significant difference in z-scored ERP peak-to-trough amplitude during fear recall in WT and *Nlgn3*^*-/y*^ rats *(p *= 0.42, F_(1, 13) _= 0.73, repeated measures two-way ANOVA, WT n = 7, KO n = 8). **H** No significant ERPs during tone habituation for WT (n = 7, *p* = 0.25, paired t-test) or *Nlgn3*^*-/y*^ (n = 8, *p* = 0.093, paired t-test) rats. Data represented as mean (solid line) ± SEM (translucent shading). **I**–**L** Significant ERPs were observed in both WT and *Nlgn3*^*−/y*^ z-scored average ERP waveforms after CS onset for tones 1–3 (WT: *p *= 0.0032, KO: *p* = 0.0099), 4–6 (WT: *p* = 0.0030, KO: *p* = 0.0084), 7–9 (WT: *p* = 0.029, KO: *p* = 0.004) and 10–12 (WT: *p* = 0.0158, KO: *p* = 0.0046, paired t-tests, WT n = 7, KO n = 8). Data represented as mean (solid line) ± SEM (translucent shading). Dotted lines represent tone onset. Data represented as mean ± SEM, dots represent individual animals.

We did not observe alterations in the excitatory synaptic properties of PAG neurons ex vivo (Fig. 5D, H), however, the synaptic inputs to neurons within the fear circuitry may still be altered in *Nlgn3*^*−/y*^ rats in vivo. Indeed, reduced freezing behaviour during fear recall and extinction has been shown to be correlated with reduced CS-evoked local-field potential (LFP) amplitudes in the PAG [75]. Therefore, we predicted CS-evoked LFPs (or “event-related potentials”, ERPs) would be reduced in *Nlgn3*^*−/y*^ rats, despite overall excitatory synaptic inputs being unchanged. We recorded LFPs in the PAG during auditory fear recall from naïve WT and *Nlgn3*^*−/y*^ rats (Fig. 6). As was seen in non-implanted animals (see Fig. 2), implanted *Nlgn3*^*−/y*^ rats showed reduced freezing behaviour during recall in comparison with WTs (Fig. 6E, F_(1,13)_ = 17.05, *p* < 0.001). *Nlgn3*^*−/y*^ rats again displayed a significantly higher response to the CS when examining immobility of the paws only in comparison with classic freezing during fear recall (Fig. 6F, *p* = 0.001, F_(1, 7)_ = 29.75), an effect that was not seen in WT animals (*p* = 0.12, F_(1, 6)_ = 3.39). WT and *Nlgn3*^*−/y*^ rats did not show any difference in paw immobility behaviour during the conditioning phase of this task (Additional file 1: Fig. S2B, *p* = 0.95, F_(1,11)_ = 0.004).

However, despite the decreased freezing behaviour of *Nlgn3*^*−/y*^ rats, we observed robust ERPs in the PAG of both *Nlgn3*^*−/y*^ and WT rats during fear recall (Fig. 6H–K), the amplitude of which did not differ between WT and *Nlgn3*^*−/y*^ rats (Fig. 6L, *p* = 0.42, F_(1, 13)_ = 0.73). It was noted, however, that the ERP peak-to-trough duration was significantly shorter in *Nlgn3*^*−/y*^ rats in comparison with WTs (Additional file 1: Fig. S7C–D, *p* = 0.042, F_(1, 13)_ = 5.09), although the biological relevance of this finding is currently unclear. We furthermore found that dPAG ERP amplitude or duration and percentage of time spent freezing was not correlated on an individual rat level (Additional file 1:  S7A, B, amplitude WT:* p* = 0.63, r = −0.22; amplitude KO: * p* = 0.41, r = −0.34; duration WT: * p*  = 0.61, r = 0.23; duration KO: * p* = 0.23, r = 0.47;  * p* 0.84, r = −0.56). In a subset of the same rats (WT n = 5, KO n = 7), we recorded LFPs during the tone habituation session to determine if ERPs were triggered by the unconditioned tone. Contrary to the ERPs seen after conditioning, no ERPs were observed during tone habituation (Fig. 6G, WT: *p* = 0.25, *Nlgn3*^*−/y*^: *p* = 0.093), and no freezing behaviour was exhibited by either genotype (Fig. 6C). These results indicate that *Nlgn3*^*−/y*^ rats display robust ERPs in the PAG during fear recall, of comparable amplitude to WTs, despite showing significantly reduced freezing behaviour. These data suggest that ERPs in the PAG reflect overall fear state elicited by the tone and are not indicative of the type of fear response behaviour (i.e. freezing, flight) exhibited.

### *Nlgn3*^*−/y*^ rats show increased jumping behaviour in response to in vivo dPAG stimulation

**Fig. 7:**
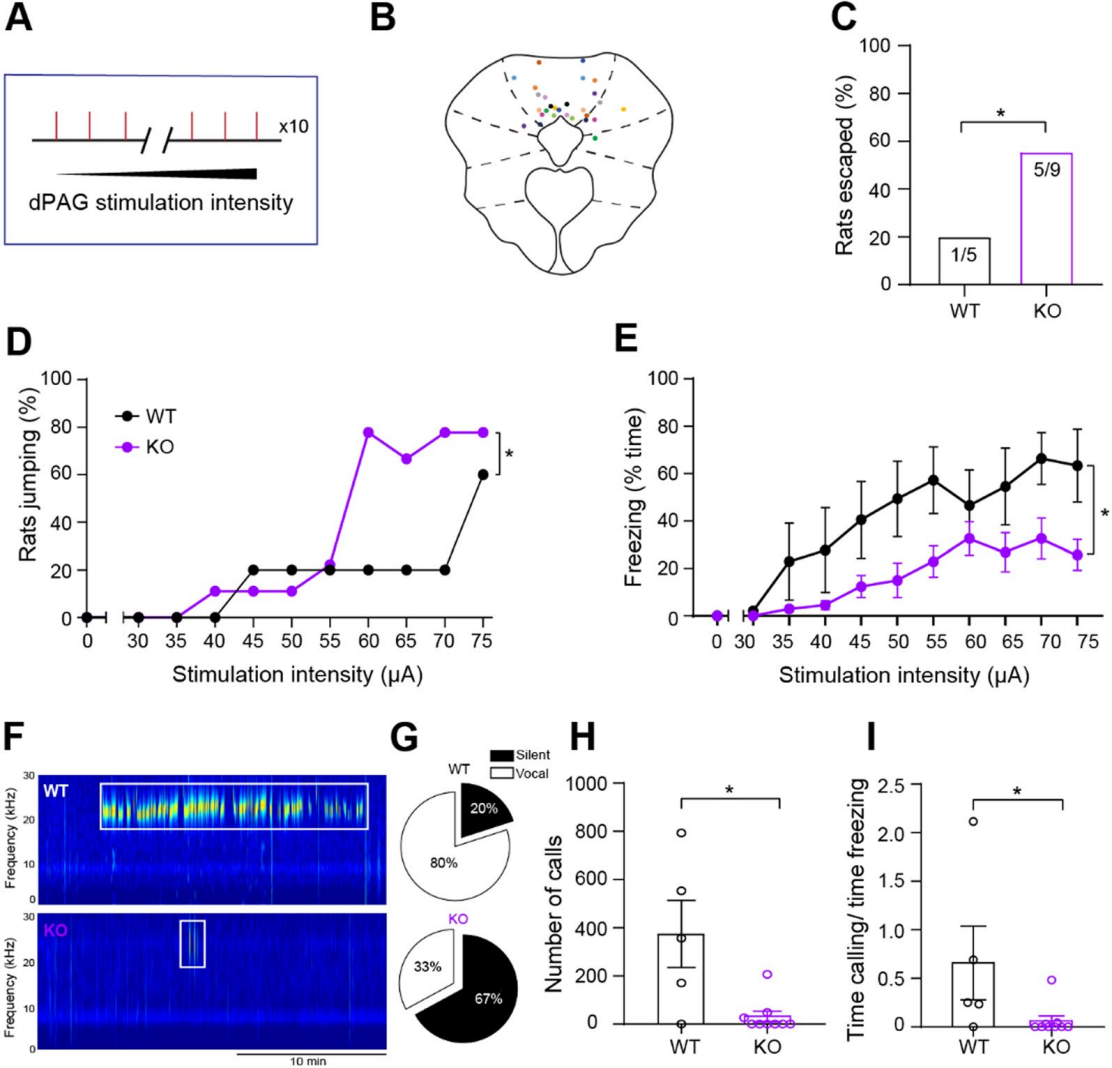
*Nlgn3*^*−/y*^ rats show increased jumping behaviour and make fewer 22 kHz calls in response to in vivo dPAG stimulation. **A** Schematic depicting dPAG stimulation protocol. **B** Location of implanted stimulating electrodes. Coloured dots represent lesion sites (bilateral) of % individual animals. **C** Significantly more *Nlgn3*^*−/y*^ rats successfully escaped the arena following dPAG stimulation in comparison with WT rats (WT n = 5, KO n = 9, *p* < 0.0001, Fisher's exact test). **D** A higher percentage of *Nlgn3*^*−/y*^ in comparison with WT rats display jumping behaviour when increasing bilateral dPAG stimulations (*p* = 0.0065, Fisher’s exact test, WT n = 5, KO n = 9). Data represented as mean **E** Classic freezing behaviour is reduced in *Nlgn3*^*−/*y^ rats (*p* = 0.025, F_(1, 12)_ = 6.58, repeated measures two-way ANOVA, WT n = 5, KO n = 9). Each data point is mean % time freezing over the entire 3-min interval following stimulation ± SEM. **F** Example spectrograms obtained from USV recordings in both WT and *Nlgn3*^*−/y*^ rats. Boxed areas indicate detected USV events. **G** Pie charts of the percentage WT and *Nlgn3*^*−/y*^ rats that were silent (emitted no USV vocalisations) or vocal during the entirety of the stimulation paradigm (30 min duration). **H** *Nlgn3*^*−/y*^ rats emit fewer USVs in the 22 kHz range compared to WT rats over the entire paradigm (*p* = 0.026, Mann–Whitney U-test = 7; n = 5 WT, 9 KO). **I** *Nlgn3*^*−/y*^ rats call less during PAG stimulation-induced freezing compared to WT (*p* = 0.034, Mann–Whitney U-test = 8; n = 5 WT, 9 KO). Data represented as mean ± SEM, clear dots represent individual animals

Several studies have reported electrical/chemical stimulation of the dPAG evokes robust escape responses such as running and jumping [4, 5, 23, 24, 60, 67, 86], followed by periods of freezing and 22 kHz ultrasonic vocalisations (USVs) [39]. Therefore, we examined whether electrical stimulation of the dPAG would promote greater flight responses in *Nlgn3*^*−/y*^ rats compared to WT controls. Bilateral dPAG stimulation (Fig. 7A) resulted in an immediate post-stimulation hyperactivity of the animals that lasted 1–5 s, followed by freezing (reviewed in [9]). A significantly higher percentage of *Nlgn3*^*−/y*^ rats escaped the arena altogether during the increasing dPAG stimulations (Fig. 7C, *p* < 0.0001), in addition to a higher percentage exhibiting jumping behaviour at dPAG stimulations of 60, 65, and 70 µA in comparison with WT rats (Fig. 7D, *p* = 0.0065). This indicates a lowered threshold for dPAG stimulation-induced flight behaviour in *Nlgn3*^*−/y*^ rats. *Nlgn3*^*−/y*^ rats also displayed reduced overall classic freezing and freezing reanalysed as immobility of paws in comparison with WT controls (Fig. 7E, *p* = 0.025, F_(1, 12)_ = 6.58, Additional file 1: Fig. S2C, *p* = 0.008, F_(1, 12)_ = 9.86). Additionally, *Nlgn3*^*−/y*^ rats produced fewer 22 kHz USVs relative to WT controls (Fig. 7F–I, *p* = 0.034).

To control for the possibility that non-specific brain stimulation causes increased escape behaviour in *Nlgn3*^*−/y*^ rats, a small cohort of animals (3 WT, 3 *Nlgn3*^*−/y*^) were bilaterally implanted with stimulating electrodes in primary somatosensory cortex (S1). Given incremental stimulation of S1, rats displayed no jumping or escape-like behaviour at any time during the behavioural assessment, irrespective of genotype. Furthermore, stimulation of S1 did not induce freezing behaviour (Additional file 1: Fig. S8). This indicates that the increase in flight behaviour in *Nlgn3*^*−/y*^ rats is specific to stimulation of the dPAG.

Together with our findings in Fig. 5B, these data support our hypothesis that increased intrinsic excitability of dPAG cells results in a circuit bias that favours flight over freezing behaviours.

## Discussion

In this study we show that the *Nlgn3*^*−/y*^ rat model of ASD/ID has distinct fear responses in both fear conditioning and as a direct result of foot-shocks. *Nlgn3*^*−/y*^ rats display increased flight and decreased freezing behaviours in response to fearful stimuli in comparison with WT controls. We also provide evidence that learning and memory are not impaired in *Nlgn3*^*−/y*^ rats. Furthermore, despite significantly reduced freezing behaviour displayed by *Nlgn3*^*−/y*^ rats during fear recall, the amplitude of tone-evoked LFPs in the PAG is unaffected. Correspondingly, excitatory synaptic inputs to cells in the PAG of *Nlgn3*^*−/y*^ rats are comparable to those of WTs. We show that dPAG cells in *Nlgn3*^*−/y*^ rats have increased intrinsic cellular excitability ex vivo, and that *Nlgn3*^*−/y*^ rats exhibit atypical responses to direct dPAG stimulation in vivo*.* To our knowledge, neither imbalance of flight–freeze responses nor electrophysiological changes in the PAG have been previously reported in any model of ASD or ID.

### *Differences in fear responses in the Nlgn3*^*−/y*^* rat model*

Fear conditioning and recall are often used to assess emotional learning in ASD/ID models, using the quantification of freezing behaviour as a proxy for the memory of the CS–US association. We find *Nlgn3*^*−/y*^ rats display less freezing behaviour (defined as no movement except for respiration) during both auditory and contextual fear recall than WT rats. Taken in isolation, these data could be interpreted as reduced fear learning and/or memory in *Nlgn3*^*−/y*^ rats. However, reanalysis of these data revealed that *Nlgn3*^*−/y*^ rats stop exploratory behaviours following onset of the tone and respond by staying fixed in the same location within space but moving the head and neck. This type of fear behaviour has been reported before in rats confronted with a snake [11, 70], and suggests *Nlgn3*^*−/y*^ rats do form an association between the CS and US, but are expressing their fear differently to WT rats. Two alternative explanations for this behaviour are a change in exploratory activity due to altered anxiety levels, or an increase in repetitive, stereotypic behaviours. However, we found no change in locomotion during open field testing, or in tests believed to reflect stereotypic behaviour (marble burying) in *Nlgn3*^*−/y*^ rats. Hence, the most parsimonious explanation for this head movement is a change in flight-related fear responses. We did not observe escape behaviour during this task, likely because the arena was fully enclosed with no possible escape route. A previous study [53] reported reduced freezing in the *Nlgn3*^*−/y*^ mouse, however no further investigation was made into the fear responses of these mice, so it is not known whether these two models of *Nlgn3* deficiency display converging phenotypes. Interestingly, a study on social interactions of *Nlgn3* R451C mice [32] reported increased jumping behaviour of these mice, consistent with our findings.

Further insight into the fear responses and learning of *Nlgn3*^*−/y*^ rats was seen in direct response to electrical foot-shocks. Active place avoidance (APA) and shock-ramp paradigms revealed *Nlgn3*^*−/y*^ rats exhibit escape behaviours in response to foot-shocks much more readily than WT controls. However, *Nlgn3*^*−/y*^ rats were able to efficiently learn the location of a shock-zone in the APA task once escape routes were blocked. Moreover, shock sensitivity testing revealed that *Nlgn3*^*−/y*^ rats are not hypersensitive to electrical shocks, but again show increased flight responses. These data further support our hypothesis that *Nlgn3*^*−/y*^ rats do not display associative learning impairments, but preferentially exhibit flight over freezing behaviour in response to fear.

### Cellular correlates of flight–freeze responses

Control of flight and freeze responses to fear are known to involve the dorsal and ventral PAG. Low-intensity electrical stimulation of the dorsal PAG has been shown to elicit freezing responses [60, 72], and higher stimulation to elicit flight responses [4, 5. 60, 67, 85, 23, 24, 72], whereas stimulation of the ventral PAG has been shown to elicit freezing responses rather than flight responses [86, 23, 24]. Correlating with the increased flight behaviour seen in *Nlgn3*^*−/y*^ rats, we observe increased intrinsic cellular excitability in the dorsal, but not ventral, PAG in slices from naïve *Nlgn3*^*−/y*^ rats. These changes in intrinsic excitability in the dorsal PAG are likely to affect the excitation/inhibition balance within the PAG, bringing the resting state of *Nlgn3*^*−/y*^ rats closer to the “threshold” of eliciting an escape response [22]. Altered inhibition could also be contributing to the freeze/flight imbalance observed in *Nlgn3*^*−/y*^ rats, however, and understanding of the relative contribution of altered excitatory and inhibitory circuitry will require a much more detailed of the fear circuit involved.

The increase in firing frequency in the dPAG of *Nlgn3*^*−/y*^ rats appears to be a result of reduced fast-afterhyperpolarisation potential (fAHP). fAHP is mediated by Ca^2+^-activated large-conductance K^+^ channels (BK) which act to hyperpolarise the membrane and reduce neuronal firing [62]. BK channel open-probabilities have been shown to be decreased in another model of ASD/ID, the *Fmr1*^*−/y*^ mouse, leading to increased neuronal excitability [19]. This presents an interesting future research avenue into the function of BK channels in the *Nlgn3*^*−/y*^ rat.

Several studies have reported a strong positive correlation between synaptic input to a neuron and LFP magnitude [1, 29, 77]. We found that miniature excitatory postsynaptic currents (mEPSCs) were not altered in either dorsal or ventral PAG cells recorded ex vivo from *Nlgn3*^*−/y*^ rat slices, suggesting that excitatory synaptic input to these PAG neurons was not altered. Consistent with this, CS-evoked LFPs (or “event-related potentials”, ERPs) recorded from the PAG during fear recall were of comparable amplitude in WT and in *Nlgn3*^*−/y*^ rats. However, we note that the peak-to-trough duration of ERPs in the dPAG of *Nlgn3*^*−/y*^ rats was significantly shorter than those in WTs. As voltage-gated ion channels have been suggested to affect LFP waveform [55, 48, 49], the altered BK channel conductance implicated by the reduced fAHP observed in dPAG neurons ex vivo may be contributing towards this phenotype. The shorter ERP we observe in the dPAG of *Nlgn3*^*−/y*^ rats during fear recall may be reflective of faster, less sustained activity in the PAG. Further experimentation is required to understand this.

ERPs recorded from the PAG during fear recall have been reported to reduce in amplitude during extinction, correlating with reduction in freezing behaviour [75]. We observe very little extinction behaviour in WT rats, however, we also observe no decrease in PAG ERP amplitude across the repeated CS presentations in WT rats. This agrees with Watson et al. [75] in that PAG ERP amplitude is associated with freezing level. However, we observe that despite exhibiting significantly less freezing behaviour than WT rats, the PAG ERP amplitudes in *Nlgn3*^*−/y*^ rats do not differ from WTs. This suggests that ERP amplitude in the PAG reflects the presence of fear, but is unrelated to the type of behavioural response the rat is exhibiting. The presence of robust amplitude ERPs in *Nlgn3*^*−/y*^ rats supports our hypothesis that these rats acquire learned fear of the tone despite the significantly reduced freezing behaviour they exhibit. It is possible that the shorter duration ERPs seen in the *Nlgn3*^*−/y*^ rats instead reflect the differences in freezing behaviour observed between genotypes.

Finally, we show that in vivo dPAG stimulation elicits flight responses in a significantly higher percentage of *Nlgn3*^*−/y*^ rats than WTs. If intrinsic excitability of dPAG neurons is increased in *Nlgn3*^*−/y*^ rats, additional stimulation of this brain region may cause flight responses to be elicited at a lower threshold than that of WT rats. Together, these results suggest that intrinsic changes within the dPAG neurons of *Nlgn3*^*−/y*^ rats underlie the preference for flight responses seen in their behaviour. In addition, compared to WT, *Nlgn3*^*−/y*^ rats emit fewer 22 kHz distress calls during freezing induced by dPAG stimulation, which further indicates potential dysfunction within the dPAG circuitry [39]. Reduced > 50 kHz calls have previously been reported in *Nlgn3*^*−/y*^ mice [53], suggesting that neuroligin-3 loss may cause altered USV emissions in both mice and rats.

## Limitations

Whilst we provide evidence that the dPAG is clearly involved in altered emotional responses in *Nlgn3*^*−/y*^ rats, the heterogenous nature of dPAG neurons hinders determination of the precise cells involved. Such a study would require retrograde labelling of subclasses of cells from specific targets of the dPAG with opto- or chemo-genetic tools. Hence, we have not identified the complete circuit by which the loss of NLGN3 and the PAG alters the balance between freeze and flight. Furthermore, we have not yet assessed the behaviour of *Nlgn3*^*−/y*^ rats using other experimental methods beside foot-shock. Utilisation of visual looming stimulus tests may provide further insight into this phenotype. A further limitation is that, whilst the human condition associated with *NLGN3* mutations appears during the first few years of life [34], we have largely focussed on phenotypes in young adult animals. Future studies will examine the developmental trajectory of *Nlgn3*^*−/y*^ rats.

## Conclusions

In conclusion, we describe altered fear responses in *Nlgn3*^*−/y*^ rats and provide evidence that this is the result of a circuit bias that predisposes flight over freeze responses. Additionally, we have shown the first phenotypic link between the PAG and ASD/ID, further study of which may provide additional insight into the mechanisms behind anxiety disorders and changes to emotional responses sometimes observed in people with ASD/ID.

## Materials and methods

### Experimental models and subject details

Sprague–Dawley *Nlgn3*^*−/y*^ transgenic rats created by Horizon Discovery, now Envigo (RRID: RGD_11568700) were housed on either a 14/10 h (Bangalore Biocluster) or 12/12 h (University of Edinburgh) light/dark cycle with a 21 ± 2 °C room temperature and food/water ad libitum. Animal husbandry was carried out by University of Edinburgh or Bangalore Biocluster technical staff. Rats were housed 4 per cage (2 WT, 2 *Nlgn3*^*−/y*^, littermates where possible) in conventional non-enriched cages, except for rats that had undergone surgeries, which were single-housed in individually ventilated cages. Body weight was monitored throughout experiments.

Experiments carried out in Edinburgh included: RNA sequencing and Western Blotting (Fig. 1), acute slice whole-cell electrophysiology recordings (Fig. 5, Additional file 1: Fig. S5), and in vivo electrophysiology and behaviour experiments (Figs. 6, 7, Additional file 1: Figs. S6–8).

Experiments carried out in Bangalore included: Western Blotting, auditory fear conditioning (Fig. 2), contextual fear conditioning (Additional file 1: Fig. S1), active place avoidance (Fig. 3, Additional file 1: Fig. S3), shock-ramp test (Fig. 4), open field (Additional file 1: Fig. S3A), marble interaction time (Additional file 1: Fig. S3E) and tail-flick test (Additional file 1: Fig. S4B).

Rats were handled for a minimum of 3 days prior to behavioural testing. Animals undergoing fear conditioning and active place avoidance tasks underwent marble burying, open field, object recognition memory tasks and three-chamber task prior to those shown in this study.

Male littermates were assigned to experimental groups based on genotype to achieve balanced cohorts. Genotyping was carried out by Transnetyx Inc. All experiments and analyses were performed blind to genotype.

### Method details

#### RNA sequencing

P60-90 male WT and *Nlgn3*^*−/y*^ rats were anaesthetised with gaseous halothane and decapitated. The brain was extracted and cooled in ice-cold (> 4 °C) carbogenated (bubbled with 95% O_2_/ 5% CO_2_) cutting artificial cerebrospinal fluid (cACSF, 87 mM NaCl, 2.5 mM KCl, 25 mM NaHCO_3_, 1.25 mM NaH_2_PO_4_, 25 mM glucose, 3.4 M sucrose, 7 mM MgCl_2_, 0.5 mM CaCl_2_) before slicing medial-prefrontal cortex. Slices were snap frozen on dry ice and stored at −80 °C.

RNA was isolated as previously described [31], and RNA integrity values determined using an Agilent 2100 Bioanalyzer and RNA 6000 Nano chips, with RIN values 8 or higher. RNA-seq libraries were prepared by Edinburgh Genomics from 1 µg total RNA using the Illumina TruSeq stranded mRNA-seq kit as per the manufacturer’s instructions. Libraries were pooled and sequenced to 50 base paired-end on the Illumina NovaSeq platform to a depth of ~ 46 million paired-end reads per sample. Reads were mapped to the rat reference genome using STAR RNA-seq aligner version 2.4.0i [20]. Read counts per gene were generated from mapped reads with featureCounts version 1.6.3 [45], using gene annotations from Ensembl version 82 [80].

#### Western blotting

P60-90 male WT and *Nlgn3*^*−/y*^ rats were anaesthetised with isoflurane and decapitated. The brain was extracted and cooled in ice-cold, carbogenated cACSF. Cortical or PAG tissue was dissected, snap frozen on dry ice, and weighed. Tissue was homogenised in ice-cold lysis buffer (150 mM NaCl, 1% Triton-X 100, 0.5% sodium deoxycholate, 0.1% SDS, 50 mM Tris (pH 8.0), protease inhibitors (Sigma), phosphatase inhibitor cocktail sets II and III (Sigma)). Samples were boiled (95 °C, 5 min) in Laemmli buffer (0.004% bromophenol blue, 10% β-mercaptoethanol, 10% glycerol, 4% SDS, 0.125 M Tris–HCl), centrifuged (16,000 G, 5 min), and vortexed.

Pierce™ BCA Protein Assay Kits (Fisher Scientific) were used to determine protein concentrations and measured using a CLARIOstar plate reader (BMG Labtech). Sample concentrations were calculated based on a bovine serum albumin standard curve (2–0.625 mg/ml).

Equal amounts of sample (20 µg total protein) along with protein ladder (PageRuler Plus Prestained Protein Ladder, Fisher Scientific, diluted in Laemmli buffer) were resolved on 10% Mini-PROTEAN TGX Precast Protein Gels (Bio-rad, 50 V 30 min, 150 V 1 h). Gels were washed in transfer buffer (Bio-rad) before transfer to nitrocellulose membranes (Bio-rad, 85 V, 2 h).

The membranes were blocked (Li-Cor buffer, 1 h) before incubation with primary antibodies (anti-NLGN3 C-terminus, Synaptic Systems, SySy-129 113, 1:1000, RRID: AB_2619816.; anti-NLGN3 N-terminus, Novus Biologicals, NBP1-90,080, 1:1000, RRID: AB_11027178) in blocking buffer with 0.01% sodium azide (10 min), then in secondary antibody (goat anti-rabbit 800, Li-Cor 1:500) in blocking buffer (2 h). After washing in TBST (TBS: Bio-rad, Tween 20: Sigma Aldrich) and TBS, membranes were imaged (Odyssey infrared, Li-COR Bioscience).

#### Behavioural paradigms

Rats aged P60-90 were used for all behaviour experiments.

##### Open field

Rats were placed inside a 60 × 60 cm arena with fresh bedding on the floor and white walls. The light intensity was uniformly ~ 20 lx. Animals were allowed to explore for 10 min before returning to their home cage. This was repeated for a total of 4 days.

##### Marble interaction task

Rats were habituated to open field (45 × 60 cm) arena with fresh bedding (2 inch) for 20 min on two consecutive days. On day 3, the rats were allowed to explore the same arena with 20 equidistantly placed opaque glass marbles (6 cm) arranged in 4 rows and 5 columns, respectively. The procedure was recorded with the overhead camera and the analysis was done using Boris v 2.98 behaviour analysis software. The light intensity throughout was uniformly maintained at 20 lx.

##### Auditory fear conditioning

Fear conditioning (context A, aluminium fear conditioning chamber with grid flooring, black/white horizontal-striped cue, and ~ 5 lx blue light) and recall (context B, 35 cm wide, 20 cm deep, 40 cm high arena with fresh bedding, mint odour, ~ 20 lx yellow light, and a transparent Perspex lid) took place in sound isolation cubicles (Coulbourn Instruments, Whitehall, Pennsylvania, USA).The behaviour of the animals was recorded using a video camera and a frame grabber (30 Hz sampling). The apparatus was cleaned with 70% ethanol before and after experiments.

Context habituation involved exploration of context B for 20 min on 2 consecutive days. On day 3, the rats were subjected to auditory fear conditioning in context A. After a baseline exploration time of 2 min, rats were presented with 3 pairings of conditioned stimulus (CS) (continuous tone, 30 s, 5 kHz, 75 dB) co-terminating with a scrambled foot-shock (unconditioned stimulus, US, 0.9 mA for 1 s, Habitest system, Coulbourn Instruments, Whitehall, Pennsylvania, USA). Each CS–US pairing was separated by inter-tone interval (ITI) of 1 min (modified from [69].

On days 4 and 5, to determine fear memory recall and extinction, rats were given 2 min to explore context B, then presented with 13 CS, with a 30 s ITI. Fear behaviour was evaluated during pre-tone, tone, and ITI.

##### Contextual fear conditioning

Rats were introduced to context A and given 2 min to explore. They were then presented with 3 unconditioned stimuli (US) pairings (0.9 mA scrambled foot-shock for 1 s), with a 90 s ITI. The following day, rats were reintroduced to context A for 10 min and fear behaviour was scored.

##### Active place avoidance

The rotating platform (Biosignal group, Brooklyn, USA) has a rectangular grid floor 100 × 100 cm) connected to a constant DC current source box for shock delivery. This was on a circular aluminium base (90 cm above ground) and run by an arena motor. A circular fence made of transparent Perspex surrounded the platform (diameter: 77 cm, height: 32 cm). For data shown in Fig. 3C–K, a transparent lid was placed on top of the circular fence. The delivery of foot-shocks (0.2 mA, 500 ms, 1500 ms interval) was tracking based (Carousel Maze Manager [3]). The 60° shock-zone was located on either North or South region and counterbalanced between rats. External to the arena, 3-dimensional cues were located at different distances from the apparatus.

Rats were held in a cabinet for 30 min before experimentation. They were habituated to the rotating arena [42] (1.5 RPM, 2 trials, 10-min interval in opaque bucket). The following day, rats were given two training sessions over two consecutive days (8 trials per session, 10-min intervals) in which the shock-zone was active. On day 4 a single probe trial was given to animals without shock-zone to assess their avoidance memory.

An overhead ceiling camera (Firewire) connected to a frame grabber (DT3155) recorded and digitised analogue video, feeding it to the tracker software (Biosignal group, Brooklyn, USA). Post-acquisition, files were analysed in Track Explorer software package (Biosignal group, Brooklyn, USA).

##### Shock-ramp test

Rats were placed within context A from the fear conditioning task. The rats were given 2.5 min to explore their environment, then were presented with 3 scrambled foot-shocks (0.06 mA, 1 s, 1.5-min intervals). After a further 1.5-min interval, a further 3 scrambled foot-shocks were given with the intensity increased to 0.1 mA (1 s, 1.5-min intervals). This was repeated with the foot-shock intensity increasing in increments (0.2, 0.3, 0.5, 0.7, 1 mA). Following this, after another 1.5-min interval the foot-shock amplitude was then dropped back to 0.1 mA and again 3 scrambled foot-shocks were given (1 s, 1.5-min intervals). Paw withdrawal, backpedalling, forward or backward running, and jumping behaviours were quantified.

##### Tail-flick test

Thermal sensitivity was assessed using tail-flick analgesia meter (Columbus Instrument). The rats were habituated to the polycarbonate restrainer for 10 min/3 days. On the 4th day, the rats were placed on the analgesia meter platform and their tail was placed in the heat slot. The heat lamp intensity was set according to the titration at various heat intensities and was fixed at 6 to get a fast response without physically damaging the tissue. Five trials were given with inter-stimulus interval of 1 min. Latency to flick the tail was documented over 5 trials.

#### In vivo recording/stimulation of the PAG

##### Implantation of local-field potential electrodes or stimulating electrodes

P60-90 rats were anesthetised with a mixture of isoflurane and O_2_ and their head shaved and sterilised. Each animal was placed on a heat-mat (37 °C) then mounted in a stereotaxic apparatus using atraumatic ear bars. Viscotears™ was applied to the eyes and 4 mg/kg Rimadyl analgesic injected subcutaneously. Surgery was then performed under aseptic conditions. Paw withdrawal reflexes were checked regularly throughout the surgery and level of isoflurane adjusted accordingly.

A midline scalp incision was made, and craniotomies performed to allow electrode implantation in the PAG (approximate coordinates: bregma −7.46 mm, ventral 4.2 mm, 1 mm lateral from midline). Recording electrodes (made in-house, ~ 0.5 mm, 140 µm diameter Teflon coated stainless-steel, A-M systems, USA) or bipolar stimulating electrodes (MS303/3-B/SP, Bilaney Ltd.) were stereotaxically lowered through the craniotomy(ies) to the PAG.

Recording electrodes were implanted unilaterally and affixed to skull using UV-activated dental cement (SpeedCem, Henry Schein), SuperBond (SunMedical, Japan), and dental cement (Simplex Rapid, Kemdent, UK) then connected to an electronic interface board (EIB 16, Neuralynx). Four screws (Screws and More, Germany) were attached to the skull for additional support and to serve as recording ground. Stimulation electrodes were implanted bilaterally and secured to the skull using the same methods as for recording. The incision was closed using absorbable surgical sutures and sterilised with iodine. Rats were left to recover for a minimum of 1 week prior to experiment start.

##### LFP recordings during fear conditioning

Recordings were made via a 16-channel digitising headstage (C3334, Intan Technologies, USA) connected to a flexible tether cable (12-pin RHD SPI, Intan Technologies, USA), custom built commutator, and Open Ephys acquisition board (OEPS, Portugal). LFP signals were bandpass-filtered from 0.1 to 600 Hz and sampled at 2 kHz in Open Ephys software. Rats implanted with LFP electrodes underwent auditory fear conditioning as described above. However, a tone habituation session of three 30 s tones (5 kHz, 75 dB, 1-min intervals) was also added before conditioning, in order to observe if ERPs were present to an unconditioned tone (NB. LFPs were only recorded during tone habituation in subset of animals (WT n = 5, KO n = 7)). Video recordings were made using Freeze Frame software (15 frames per second, Actimetrics) synchronised with electrophysiological signals using TTL pulses.

##### In vivo PAG stimulation

Rats implanted with stimulating electrodes were placed inside context B arena as described for the fear conditioning paradigm. Rats were allowed to explore the arena for 2 min, then stimulation (0.1 ms pulses, 100 Hz, 2 s) began at an intensity of 30 μA (DS3 isolated constant current stimulators, Digitimer Ltd.) and increased in 5 μA steps up to a maximum of 75 μA [39], with intervals of 3 min. Behavioural responses were recorded throughout the protocol using Freeze Frame software.

A M500-384 USB Ultrasound Microphone ultrasound detector positioned above the stimulation arena coupled to BatSound Touch Lite (Pettersson Elektronik) was used to record USVs. Recordings were sampled at 384 kHz, with a spectrogram window size of 512.

##### Histology

Following behavioural testing, rats implanted with recording or stimulating electrodes were anesthetised with gaseous isoflurane and intraperitoneal injection of pentobarbital (27.5 mg/kg) until hindpaw reflexes were absent. A current pulse of 100 µA for 2 s (DS3 isolated constant current stimulators, Digitimer Ltd.) was passed through the headstage to lesion electrode sites. Rats were then transcardially perfused with phosphate-buffered saline, followed by 4% paraformaldehyde (PFA). The brains were extracted and left in 4% PFA for 24 h. Brains were then cut into 80 µm sections on a vibratome or freezing microtome, and these sections mounted onto glass slides. Sections were then stained with cresyl violet acetate, covered with DPX mounting medium and coverslipped. A Leica DMR upright bright-field microscope was used to image the lesion site. Location of the lesion site was projected onto a schematic of the PAG.

#### Ex vivo whole-cell patch-clamp recordings

Acute brain slices were made from rats aged 4–6 weeks (or 8–10 weeks, Additional file 1: Fig. S6 only), as previously described [8]. The brain was quickly extracted and cooled in ice-cold (> 4 °C) carbogenated (95% O_2_/5% CO_2_) cACSF. The cerebellum was removed, and the brain cut coronally in half before slicing the PAG coronally at 0.05 mm/s into 400 µm slices on a Leica VT 1200S vibratome. Slices were allowed to recover in carbogenated cACSF at 35 ± 1 °C for 30 min, and then stored at room temperature until recording.

##### Whole-cell recordings

Slices were transferred to a recording chamber where they were perfused with carbogenated recording-ACSF (125 mM NaCl, 2.5 mM KCl, 25 mM NaHCO_3_, 1.25 mM NaH_2_PO_4_, 25 mM glucose, 1 mM MgCl_2_, 2 mM CaCl_2_) at 31 ± 1 °C at a rate of 3–6 ml/min. Slices were visualised using infrared differential interference contrast (IR-DIC) video microscopy, using a digital camera (DAGE-MTI) mounted on an upright microscope (U-CA, Olympus, Japan) and a 40 × water immersion objective was used for all experiments. These were paired with Scientifica slicescope, patchstar and heater units and controlled using LinLab 2 (Scientifica).

Electrodes with 3–6 MΩ tip resistance were pulled from borosilicate glass capillaries (1.7 mm outer/1 mm inner diameter, Harvard Apparatus, UK) horizontal electrode puller (P-97, Sutter Instruments, CA, USA). A potassium-gluconate based internal solution (120 mM K-gluconate, 20 mM KCl, 10 mM HEPES, 4 mM NaCl, 4 mM Mg_2_ATP, 0.3 mM Na_2_GTP, pH 7.4, 290–310 mOsm) was used for all current-clamp recordings. A caesium-gluconate based internal solution (140 mM Cs-gluconate, 3 mM CsCl, 10 mM HEPES, 0.2 mM EGTA, 5 mM QX-314 chloride, 2 mM MgATP, 0.3 mM Na_2_GTP, 2 mM NaATP, 10 mM phosphocreatine, pH 7.4, 290–310 mOsm) was used for all voltage-clamp recordings.

Cells in the dorsal and ventral PAG were identified by area. A −70 mV holding potential was applied following the creation of a > 1 GΩ seal. The fast and slow membrane capacitances were neutralised before breaking through the cell membrane to achieve whole-cell configuration. For mEPSC recordings, gap-free recordings were performed in voltage-clamp configuration for 10 min in the presence of picrotoxin (50 µM) and tetrodotoxin (300 nM). Cells were discarded if access resistance was > 30 MΩ or changed by > 20%. Intrinsic property recordings were carried out in current-clamp configuration, as follows. Resting membrane potential (RMP) of the cell was recorded with current clamped at 0 pA, and all other protocols recorded with appropriate current injection to hold the cell at −70 mV. Cells were discarded if RMP was more depolarised than −40 mV or if access resistance was > 30 MΩ or changed by > 20%. Input resistance and membrane time constant were assessed by injecting a −10 pA step, and cell capacitance calculated from these values. Input–output curves and rheobase potential were assessed by current injections of −200 to + 100 pA for 500 ms (10 pA steps). Action potential kinetics were gleaned from the rheobase action potential. Recordings were made using a Multiclamp 700B amplifier linked to pCLAMP™ Clampex software (Molecular Devices). Signals were sampled at 20 kHz (Digidata1440 or Digidata1550A, Molecular Devices) and Bessel-filtered at 2 kHz for voltage-clamp recordings and 10 kHz for current-clamp recordings.

### Quantification and statistical analysis

Fear behaviour was scored as either “classic freezing”, defined as no movement except for respiration [7], or “paw immobility response”, defined as all 4 paws unmoving, however allowing for movement of the head and neck. Behaviours were scored if lasting > 1 s. For the shock-ramp paradigm, paw withdrawal responses, backpedalling, forward/backward running, and jumping were scored. For dPAG stimulation experiments, response behaviours were scored as freezing, startle, attention, running, or jumping, according to criteria described previously [11]. All behaviour was manually scored using BORIS [27] or in-house software Z-score (created by O. Hardt).

Stimfit software [28] combined with custom-written Matlab scripts (A. Jackson) were used for whole-cell patch-clamp data analysis. mEPSCs were analysed for the final 3 min of the 10-min recording. Events were detected using template-matching and filtered to 3 × standard deviation of baseline [14].

Data collected from LFP recordings were analysed using custom-written MATLAB scripts (F. Inkpen, A. Jackson). Raw traces of 3 tones were averaged, and then z-scored to normalise data to baseline noise. Peak and trough of the LFPs were manually selected.

Raven Lite software (Cornell Lab, Centre for Conservation Bioacoustics) was used to generate spectrograms and to manually quantify USVs.

Throughout, all data is shown as mean ± SEM, or as percentages where appropriate. Statistics were carried out using GraphPad Prism software 8.0, SPSS, or RStudio. Two-way ANOVAs with Holm–Sidak post hoc repeated measures test (Figs. 2, 3, 4, 5, 6, 7, Additional file 1: Figs. S1, 2, 3, 5–7), Pearson’s R correlation (Additional file 1: Fig. S7), unpaired t-tests (Fig. 4, Additional file 1: Fig. S4), paired t-tests (Additional file 1: Fig. S4), Fisher’s exact tests (Figs. 3, 7), three-way ANOVAs (Figs. 2, 6, Additional file 1: Fig. S1), or generalised linear mixed modelling (GLMM) (Fig. 5, Additional file 1: Fig. S5) were employed. N was taken to be animal average in all cases to avoid pseudoreplication, except for when GLMM statistical analysis was employed. R packages lme4 and car were utilised to perform GLMMs. *p* values of < 0.05 were taken to be significant, and one star (*) represents all *p* values < 0.05 throughout. Full details of statistical tests and results are described in Additional file 2: Tables S1, 2.

## Supplementary Information

**Additional file 1:** **Supplemental figures S1-S9. Figure S1.** *Nlgn3*^*−/y*^ rats display reduced classic freezing behaviour in a contextual fear conditioning paradigm. **Figure S2.** Freezing analysed as “paw immobility response” (all four paws unmoving but allowing for movement of head and neck). **Figure S3.** WT and *Nlgn3*^*−/y*^ rats show similar activity in an open field, rotational platform & marble interaction tasks. **Figure S4.** Effect of repeated footshocks & thermal stimulus on WT and *Nlgn3*^*−/y*^ rats. **Figure S5.** Intrinsic properties of PAG cells recorded from WT and *Nlgn3*^*−/y*^ rats. **Figure S6.** Hyperexcitability of dorsal, but not ventral PAG neurons in 8-10 week old *Nlgn3*^*−/y*^ rats. **Figure S7.** PAG LFPs during fear recall are significantly shorter duration in *Nlgn3*^*−/y*^ rats. **Figure S8.** Defensive reactions were not elicited by electrical stimulation of primary somatosensory cortex in WT or *Nlgn3*^*−/y*^ rats. **Figure S9.** Western blots showing lack expression of NLGN3 in *Nlgn3*^*−/y*^ rats both in sensory cortex and periaqueductal grey.

**Additional file 2: Supplemental table 1.** Summary of statistics for main figures 2–7. **Supplemental table 2.** Summary of statistics for supplemental figures 1–7.

## Data Availability

The datasets generated and/or analysed during the current study are available from the corresponding author on reasonable request.
