# Supplementary material for: Imbalance of flight–freeze responses and their cellular correlates in the Nlgn3−/y rat model of autism
Source: Mol Autism. 2022 Jul 18;13:34. doi: 10.1186/s13229-022-00511-8 (PMC9290228; doi:10.1186/s13229-022-00511-8)
Supplement: Supplementary file 1 — Additional file 1: Supplemental figures S1-S9. Figure S1. Nlgn3−/y rats display reduced classic freezing behaviour in a contextual fear conditioning paradigm. Figure S2. Freezing analysed as “paw immobility response” (all four paws unmoving but allowing for movement of head and neck). Figure S3. WT and Nlgn3−/y rats show similar activity in an open field, rotational platform & marble interaction tasks. Figure S4. Effect of repeated footshocks & thermal stimulus on WT and Nlgn3−/y rats. Figure S5. Intrinsic properties of PAG cells recorded from WT and Nlgn3−/y rats. Figure S6. Hyperexcitability of dorsal, but not ventral PAG neurons in 8-10 week old Nlgn3−/y rats. Figure S7. PAG LFPs during fear recall are significantly shorter duration in Nlgn3−/y rats. Figure S8. Defensive reactions were not elicited by electrical stimulation of primary somatosensory cortex in WT or Nlgn3−/y rats. Figure S9. Western blots showing lack expression of NLGN3 in Nlgn3−/y rats both in sensory cortex and periaqueductal grey. [file 13229_2022_511_MOESM1_ESM.pdf]

Additional file 1.Supplemental figures S1-S9. Figure S1. Nlgn3$^{-/y}$ rats display reduced classic freezing behaviour in a contextual fear conditioning paradigm. Figure S2. Freezing when analysed as "paw immobility response" (all four paws unmoving but allowing for movement of head and neck). Figure S3. WT and Nlgn3$^{-/y}$ rats show similar activity in an open field, rotational platform & show no repetitive interaction with marbles in marble burying task. Figure S4. Effect of repeated footshocks & thermal stimulus on WT and *Nlgn3*$^{-/y}$ rats. Figure S5. Intrinsic properties of PAG cells recorded from WT and *Nlgn3*$^{-/y}$ rats. Figure S6. Hyperexcitability of dorsal, but not ventral PAG neurons in 8-10 week old *Nlgn3*$^{-/y}$ rats. Figure S7. PAG LFPs during fear recall are significantly shorter duration in *Nlgn3*$^{-/y}$ rats. Figure S8. Defensive reactions were not elicited by electrical stimulation of primary somatosensory cortex in WT or *Nlgn3*$^{-/y}$ rats. Figure S9. Western blots showing lack expression of NLGN3 in *Nlgn3*$^{-/y}$ rats both in sensory cortex and periaqueductal grey.

Figure S1

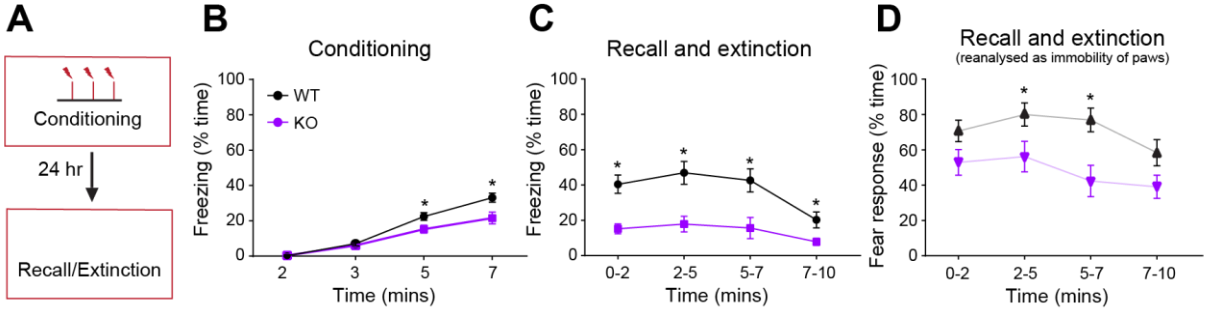

Supplemental Figure 1. *Nlgn3*$^{-/y}$ rats display reduced classic freezing behaviour in a contextual fear conditioning paradigm. (A) Schematic of contextual fear conditioning paradigm. (B) Classic freezing behaviour is reduced in *Nlgn3*$^{-/y}$ rats in comparison to WTs during the conditioning phase of contextual fear conditioning ($p = 0.025$, $F_{(1, 25)} = 5.67$, repeated measures two-way ANOVA, WT n = 13, KO n = 14). (C) Classic freezing behaviour is reduced in *Nlgn3*$^{-/y}$ rats in comparison to WTs during the recall phase of contextual fear conditioning ($p < 0.0001$, $F_{(1, 25)} = 26.61$, repeated measures two-way ANOVA, WT n = 13, KO n = 14). (D) When analysed as "immobility response" (i.e all four paws unmoving but allowing for movement of head and neck, shown in light purple/grey) *Nlgn3*$^{-/y}$ rats show a response to the CS significantly different to classic freezing (main effects of scoring method: $p < 0.0001$, $F_{(1, 25)} = 200.82$, and genotype: $p < 0.0001$, $F_{(1, 25)} = 20.65$, three-way ANOVA, WT n = 13, KO n = 14).

Data represented as mean ± SEM.

Figure S2

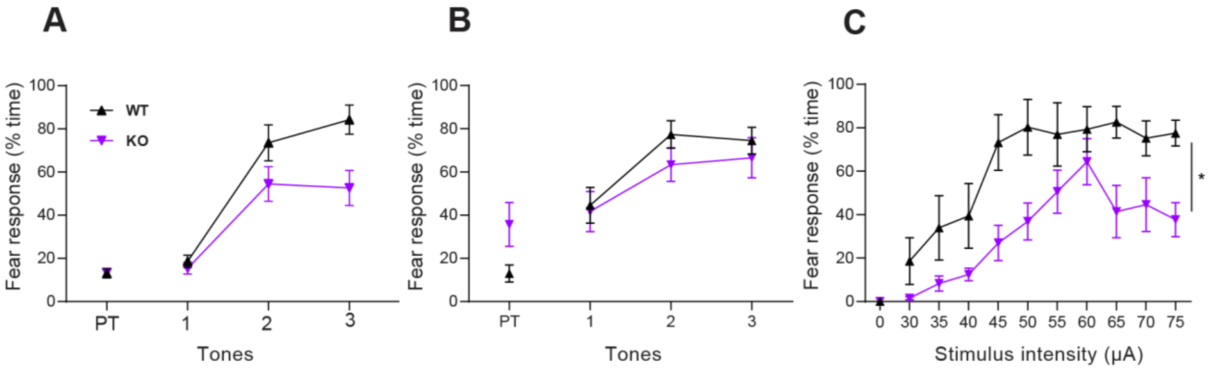

Supplementary Figure 2: Freezing when analysed as "paw immobility response" (all four paws unmoving but allowing for movement of head and neck). (A) *Nlgn3*$^{-/y}$ rats display less paw immobility response compared to WT rats during conditioning phase of auditory fear conditioning task (p = 0.008, $F_{(1, 22)}$ = 8.333, repeated measures two-way ANOVA, WT n = 12, KO n = 12). (B) *Nlgn3*$^{-/y}$ rats show similar paw immobility levels compared to WT rats during conditioning phase of auditory conditioning task in field recording electrode implanted rats (p = 0.95, $F_{(1,11)}$ = 0.004, repeated measures two-way ANOVA, WT n = 5, KO n = 8). (C) Percentage time exhibiting paw immobility response is reduced in *Nlgn3*$^{-/y}$ rats during dPAG stimulation (p = 0.008, $F_{(1, 12)}$ = 9.86, repeated measures two-way ANOVA, WT n = 5, KO n = 9). Data represented as mean ± SEM.

Figure S3

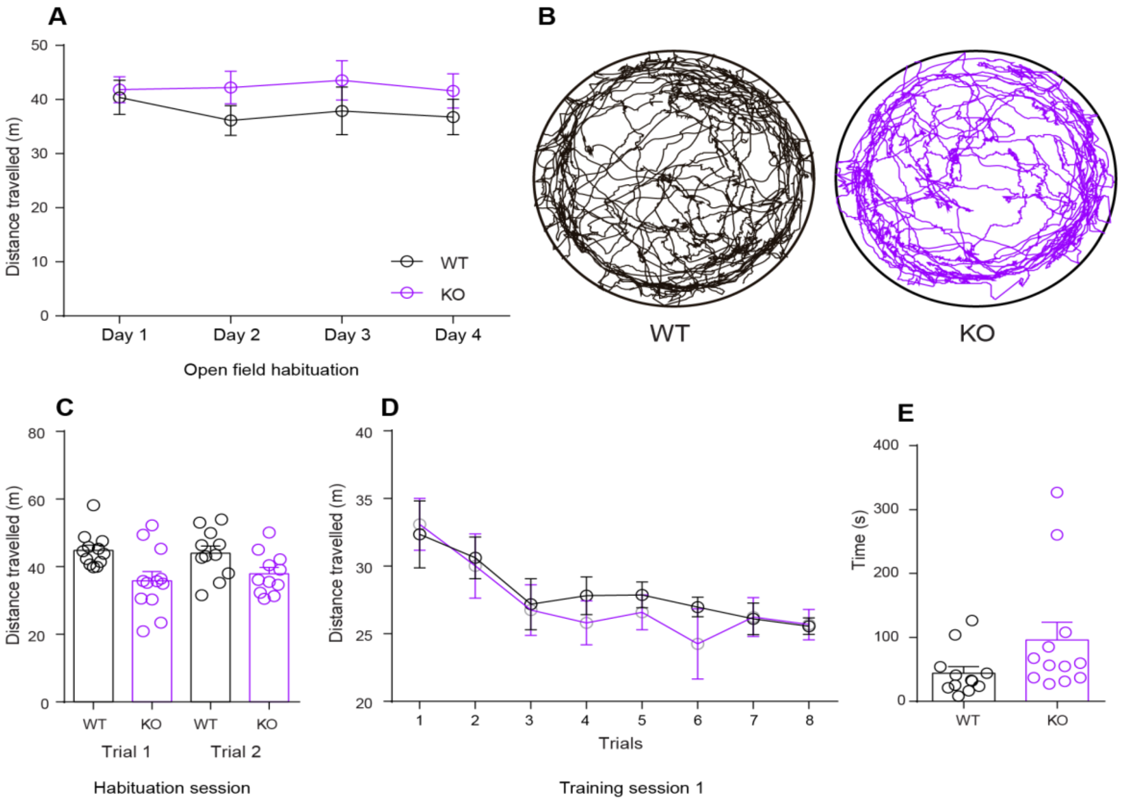

Supplemental Figure 3. WT and *Nlgn3*$^{-/y}$ rats show similar activity in an open field, rotational platform & show no repetitive interaction with marbles in marble burying task. (A) Distance travelled of WT and *Nlgn3*$^{-/y}$ rats during 4 days of open field testing (p = 0.29, $F_{(1, 22)}$ = 1.19, repeated measures two-way ANOVA, WT n = 12, KO n = 12). (B) Representative track plots from WT and *Nlgn3*$^{-/y}$ rats during habituation to the rotational platform. (C) Distance travelled is not different between WT and *Nlgn3*$^{-/y}$ rats during habituation to the rotational platform (Trial 1 WT vs *Nlgn3*$^{-/y}$, p = 0.99 & Trial 2 WT vs *Nlgn3*$^{-/y}$ p = 0.89, one way ANOVA, WT n = 12, KO n = 11). (D) Distance travelled is not different between WT and *Nlgn3*$^{-/y}$ rats during training session 1 of APA task (p = 0.59, $F_{(1, 21)}$ = 0.29, repeated measures two-way

ANOVA, WT n = 12, KO n = 11). (E) Time spent in interaction with marbles is not different between WT and *Nlgn3*$^{-/y}$ in marble burying task (p = 0.09, unpaired t-test, WT n = 12, KO n = 12).

Data represented as mean ± SEM.

Figure S4

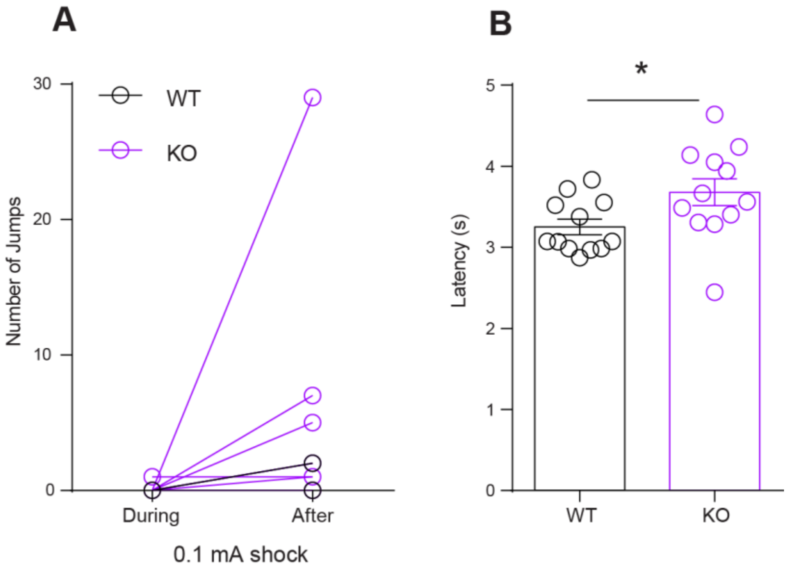

Supplemental Figure 4. Effect of repeated footshocks & thermal stimulus on WT and *Nlgn3*$^{-/y}$ rats. (A) Number of jumps exhibited in response to 0.1 mA foot-shocks during (following 0.06 mA) and after (following 1 mA) shock ramp testing. Number of jumps are not significantly different for WT (p = 0.35, paired t-test, n = 11) or KO (p = 0.10, paired t-test, n = 14) animals. (B) Tail-flick latency is significantly not different between WT and *Nlgn3*$^{-/y}$ rats during thermal tail flick test (p = 0.036, unpaired t-test, WT n = 12, KO n = 12).

Dots represent individual animals.

Figure S5

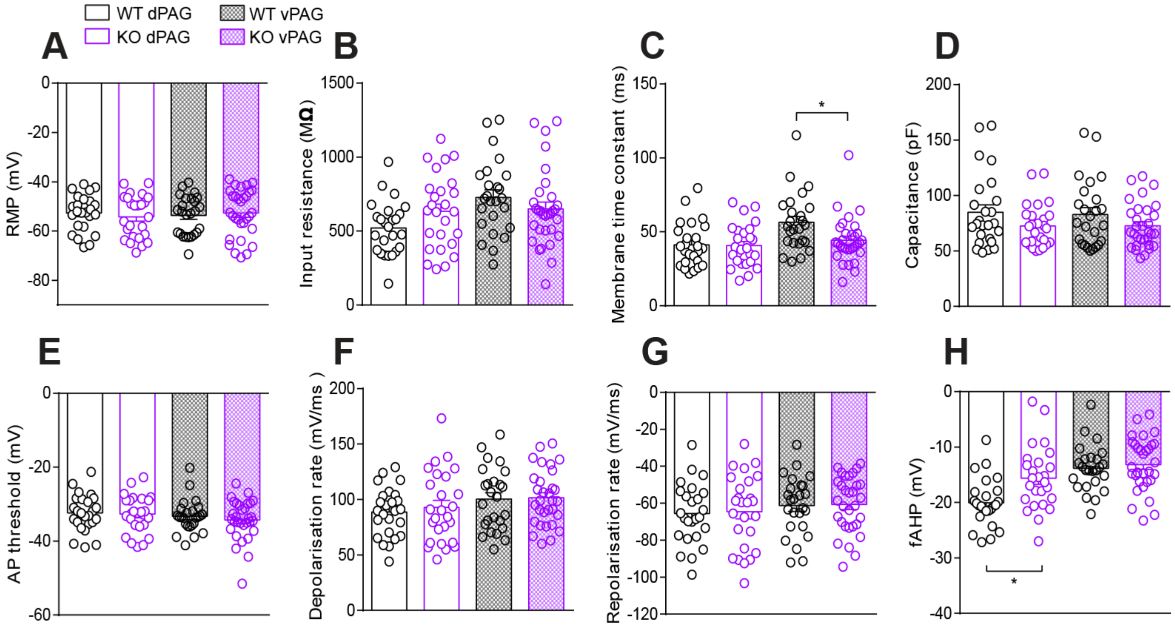

Supplemental Figure 5. Intrinsic properties of PAG cells recorded from WT and *Nlgn3*$^{-/y}$ rats.
(A) Resting membrane potential is comparable between *Nlgn3*$^{-/y}$ and WT rats in both dPAG (p = 0.61, GLMM, dPAG WT, 25 cells/ 10 rats, dPAG KO 26 cells/ 9 rats) and vPAG cells (p = 0.75, GLMM, WT 24 cells/10 rats, vPAG KO 28 cells/ 9 rats). (B) Input resistance is comparable between *Nlgn3*$^{-/y}$ and WT rats in both dPAG (p = 0.090, GLMM, dPAG WT, 25 cells/ 10 rats, dPAG KO 26 cells/ 9 rats) and vPAG cells(p = 0.26, GLMM, vPAG WT 24 cells/ 9 rats, vPAG KO 28 cells/ 10 rats). (C) Membrane time constant is comparable between *Nlgn3*$^{-/y}$ and WT rats in cells recorded from dPAG (p = 0.78, GLMM, dPAG WT, 25 cells/ 10 rats, dPAG KO 26 cells/ 9 rats), however is reduced in vPAG cells of *Nlgn3*$^{-/y}$ compared to WT (p = 0.0095, GLMM, vPAG WT 24 cells/ 9 rats, vPAG KO 28 cells/ 10 rats). (D) Capacitance is comparable between *Nlgn3*$^{-/y}$ and WT rats in both dPAG (p = 0.11, GLMM, dPAG WT, 25 cells/ 10 rats, dPAG KO 26 cells/ 9 rats) and vPAG cells (p = 0.19, GLMM, vPAG WT 24 cells/ 9 rats, vPAG KO 28 cells/ 10 rats). (E) Action potential (AP) threshold is comparable between *Nlgn3*$^{-/y}$ and WT rats in both dPAG (p = 0.86, GLMM, dPAG WT, 25 cells/ 10 rats, dPAG KO 26 cells/ 9 rats) and vPAG cells (p = 0.47, GLMM, vPAG WT 24 cells/ 9 rats, vPAG KO 28 cells/ 10 rats). (F) No difference in AP depolarisation rate between WT and *Nlgn3*$^{-/y}$ rats in either dPAG (p = 0.71, GLMM, dPAG WT, 25 cells/ 10 rats, dPAG KO 26 cells/ 9 rats) or vPAG cells (p = 0.90, GLMM, vPAG WT 24 cells/ 9 rats, vPAG KO 28 cells/ 10 rats). (G) No difference in AP repolarisation rate between WT and *Nlgn3*$^{-/y}$ rats in either dPAG (p = 0.76, GLMM, dPAG WT, 25 cells/ 10 rats, dPAG KO 26 cells/ 9 rats) or vPAG cells (p = 0.90, GLMM, vPAG WT 24 cells/ 9 rats, vPAG KO 28 cells/ 10 rats). (H) Fast afterhyperpolarisation potential (fAHP) is significantly reduced in *Nlgn3*$^{-/y}$ rat dPAG neurons in comparison to WT (p = 0.0047, GLMM, dPAG WT, 25 cells/ 10 rats, dPAG KO 26 cells/ 9 rats) but unchanged in vPAG neurons (p = 0.58, GLMM, vPAG WT 24 cells/ 9 rats, vPAG KO 28 cells/ 10 rats).

Data represented as mean ± SEM, dots represent individual cells.

Figure S6

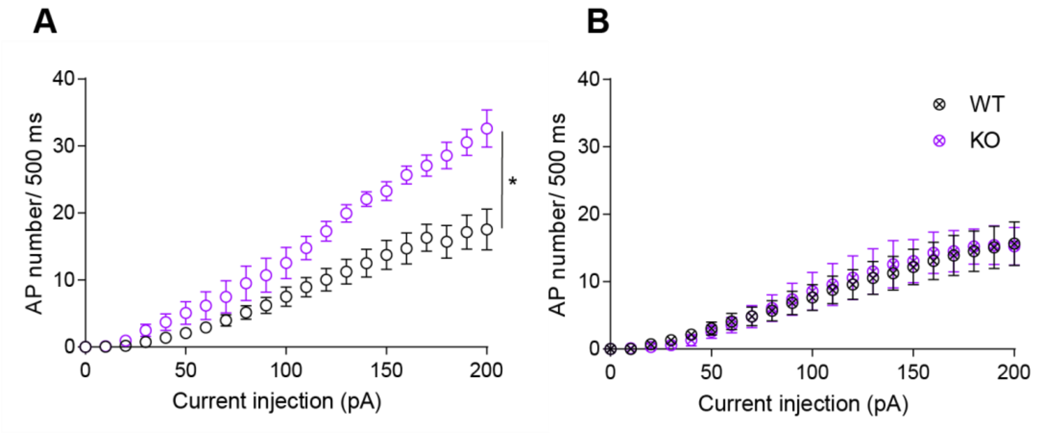

Supplemental Figure 6. Hyperexcitability of dorsal, but not ventral PAG neurons in 8-10 week old *Nlgn3*$^{-/y}$ rats. (A) dPAG cells from 8-10 week old *Nlgn3*$^{-/y}$ rats fire an increase number of action potentials in response to increasing current injections in comparison to WT ($p = 0.0094$, $F_{(1, 9)} = 10.82$, WT n = 15 cells/ 7 rats, KO n = 6 cells/ 4 rats). (B) dPAG cells from 8-10 week old WT and *Nlgn3*$^{-/y}$ rats fire an equivalent number of action potentials in response to increasing current injections ($p = 0.92$, $F_{(1, 13)} = 0.0097$, WT n = 14 cells/ 7 rats, KO n = 6 cells/ 4 rats).

Data represented as animal mean ± SEM.

Figure S7

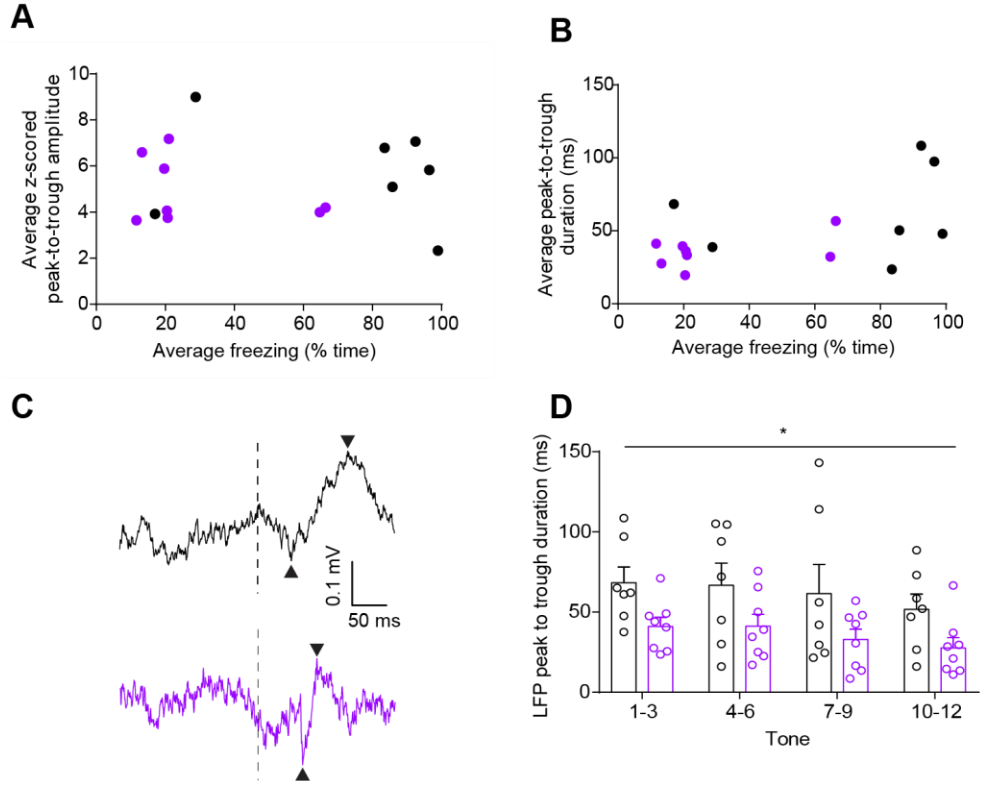

Supplemental Figure 7. PAG LFPs during fear recall are significantly shorter duration in *Nlgn3*$^{-/y}$ rats. (A) Average freezing behaviour and ERP amplitude do not correlate (WT: $p = 0.63$, $r = -$

0.22 n = 7, Pearson's R, KO: p = 0.41, r = -0.34, n = 8). (B) Average freezing behaviour and ERP duration do not correlate correlation (WT: p = 0.61, r = 0.23, Pearson's R, n = 7, KO: p = 0.23, r = 0.47, Pearson's R, n = 8). (C) Example LFP traces from WT (black) and *Nlgn3*$^{-/y}$ (purple) rats. Black arrows denote trough and peak. (D) *Nlgn3*$^{-/y}$ rats display significantly faster tone-evoked LFPs in the PAG during fear recall in comparison to WT rats (p = 0.042, $F_{(1, 13)}$ = 5.09, two-way ANOVA, WT n = 7, KO n = 8).

Data represented as mean ± SEM, dots represent individual animals.

Figure S8

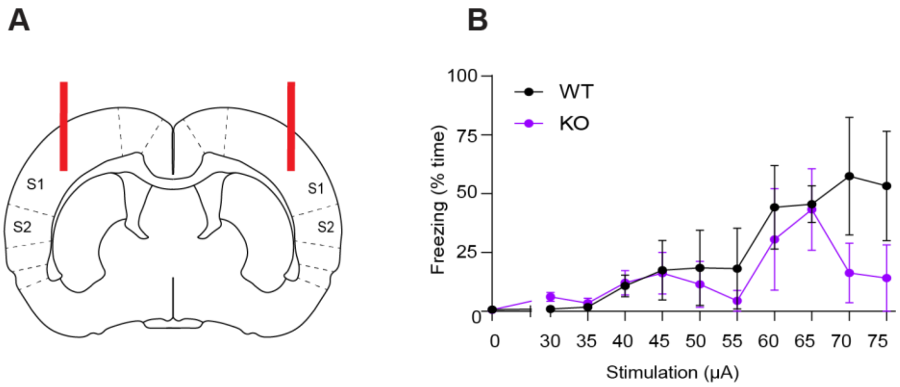

Supplemental figure 8. Defensive reactions were not elicited by electrical stimulation of primary somatosensory cortex in WT or *Nlgn3*$^{-/y}$ rats. (A) Schematic depicting stimulating electrode (red lines) implant site. (B) Freezing behaviour, defined as no movement except for respiration, for 3 WT and 3 *Nlgn3*$^{-/y}$ rats receiving cortical stimulation. Resting or sleeping was indistinguishable from freezing given this definition.

Data represented as mean ± SEM, points represent average freezing time for 3 minutes post-stimulation.

Figure S9

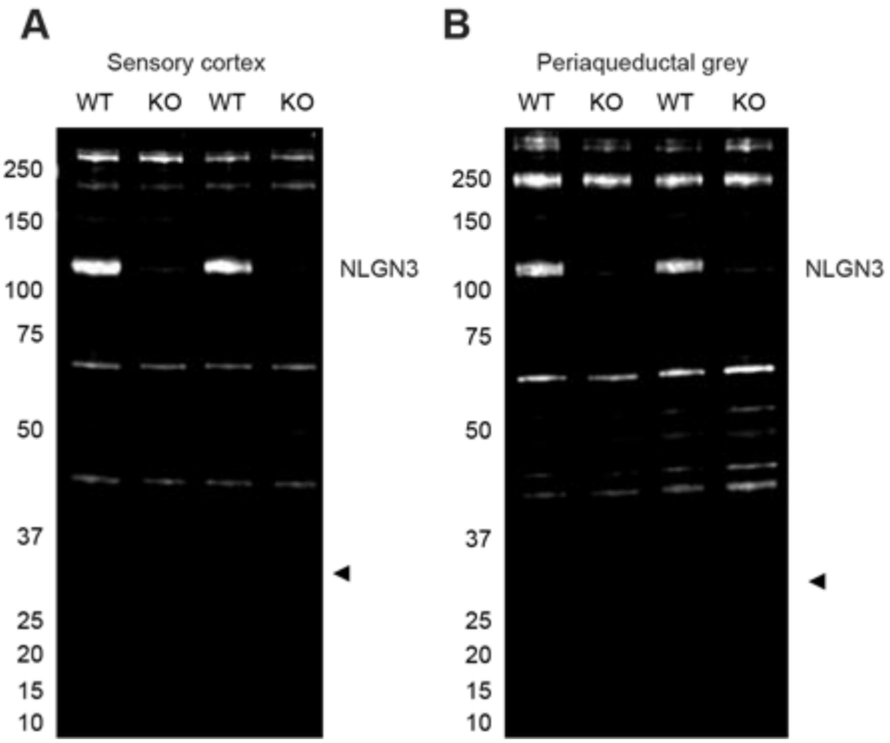

Supplemental figure 9. Western blots showing lack expression of NLGN3 in *Nlgn3*$^{-/y}$ rats both in sensory cortex and periaqueductal grey. Representative western blot of cortical **(A)** and periaqueductal grey **(B)** of WT and *Nlgn3*$^{-/y}$ tissue using anti-NLGN3 antibody. No NLGN3 protein was found in *Nlgn3*$^{-/y}$ rats (WT n = 4, KO n = 4).
