# Supplementary material for: Imbalance of flight–freeze responses and their cellular correlates in the Nlgn3−/y rat model of autism
Source: Mol Autism. 2022 Jul 18;13:34. doi: 10.1186/s13229-022-00511-8 (PMC9290228; doi:10.1186/s13229-022-00511-8)
Supplement: Supplementary file 2 — Additional file 2: Supplemental table 1. Summary of statistics for main figures 2–7. Supplemental table 2. Summary of statistics for supplemental figures 1–7. [file 13229_2022_511_MOESM2_ESM.docx]

| **Supplementary table 1. Statistics of figures.** | | | | |
| --- | --- | --- | --- | --- |
| **Figure** | **Task/Measure** | **Population size** | **Statistical test** | **Results/Comparison** |
| 2B | Auditory fear conditioning (Conditioning) | WT=12  KO=12 | Two-way repeated measure ANOVA | p<0.0001,  F _(1, 22)_=6.61 |
| 2C | Auditory fear conditioning (Recall and extinction 1) |  |  | p=0.001,  F _(1, 22)_=13.36 |
| 2D | Auditory fear conditioning (Recall and extinction 2) |  |  | p=0.0009,  F _(1, 22)_=14.61 |
| 2E | Auditory fear conditioning (Recall and extinction 1; reanalysed as immobility of paws) |  | Three-way ANOVA | Scoring F_(1, 22)_ = 20.32, p < 0.0001; tone F_(12, 264)_ = 18.75, p <0.0001; genotype F_(1, 22)_ = 15.85, p = 0.001; scoring x genotype F_(1, 22)_ = 0.61, p = 0.012; tone x genotype F_(12, 264)_ = 1.19, p < 0.0001; scoring x tone F_(12, 264)_ = 0.49, p = 0.92, scoring x tone x genotype F_(12, 264)_ = 3.23, p < 0.0001  **Post hoc two-way ANOVAs:**  WT _(classic)_ vs KO _(classic)_:  Tone F _(12,264)_ =12.52, p<0.0001; tone x genotype F _(12,264)_ =3.871, p<0.0001; genotype F _(1, 22)_ =13.36, p<0.001  WT _(Paw immobility)_ vs KO _(Paw immobility)_:  Tone F _(12,264)_ =12.33, p<0.0001; tone x genotype F _(12,264)_ =0.61, p=0.83; genotype F _(1,22)_=12.1, p<0.002  WT _(classic)_ vs WT _(Paw immobility)_  Scoring F _(1, 11)_ =7.58, p<0.019; tone F _(12,132)_ =12.16, p<0.0001; scoring x tone F _(12,132)_=0.56, p=0.7  KO _(classic)_ vs KO _(Paw immobility)_  Scoring F _(1, 11)_ =13.30, p<0.004; tone F _(12,132)_ =7.43, p<0.0001; scoring x tone F _(12,132)_ =5.69, p<0.0001 |
| 2F | Classic freezing Vs immobility of paws during recall |  | Three-way ANOVA | Scoring F_(1,22)_=29.89 , p<0.0001; time F_(1,22)_=191.25; genotype F_(1,22)_=15.21, p=0.001; scoring x genotype F_(1,22)_=8.49, p=0.007; time x genotype F_(1,22)_=19.33, p<0.0001; scoring x tone F_(1,22)_=15.59 p=0.001, scoring x tone x genotype F_(1,22)_=7.5, p=0.012  **Post hoc test: Bonferroni-corrected paired t-tests:**  WT pretone_(classic)_ vs WT CS-response_(classic)_ p<0.0001  WT pretone _(paw immobility)_ vs WT CS-response_(paw immobility)_ p<0.0001  KO pretone_(classic)_ vs KO CS-response_(classic)_ p=0.008  KO pretone_(paw immobility)_ vs KO CS-response _(paw immobility)_ p<0.0001  WT CS-response_(classic)_ vs WT CS-response_(paw immobility)_ p=0.24  KO CS-response_(classic)_ vs KO CS-response_(paw immobility)_ p<0.0001 |
| 3B | Percentage of escape in rotating arena | WT=9  KO=9 | Fischer exact test | p=0.0034 |
| 3E | Shock zone entry (Training session1) | WT=12  KO=11 | Two-way repeated measure ANOVA | p=0.0045,  F _(1, 21)_ = 10.09 |
| 3F | Time in shock zone (Training session 1) | WT=12  KO=11 |  | p=0.027,  F _(1, 21)_ = 5.68 |
| 3G | Shock zone entry (Training session 2) |  |  | p=0.044,  F _(1, 21)_ = 4.6 |
| 3H | Time in shock zone (Training session 2) |  |  | p=0.025,  F _(1, 21)_ = 5.8 |
| 3J | Shock zone entry (Probe) |  |  | p=0.0039,  F _(1, 21)_ = 10.51 |
| 3K | Time in shock zone (Probe) |  |  | p=0.045,  F _(1, 21)_ = 4.53 |
| 4B | Response to shock | WT=11  KO=14 | Unpaired t-test | p=0.13 |
| 4C | Backpedalling response to shock |  | Unpaired t-test | p=0.26 |
| 4D | Number of jumps |  | Two-way repeated measure ANOVA | p=0.0081,  F _(1, 23)_ = 8.39 |
| 5B | dPAG Action potential number | WT n=25 cells/10 rats  KO n=26 cells/9 rats | Two-way repeated measure ANOVA | p=0.018,  F _(1, 17)_ = 6.87 |
| 5C | dPAG Rheobase potential | WT n=25 cells/10 rats  KO n=26 cells/9 rats | GLMM | p=0.014, |
| 5D | dPAG mEPSC amplitude and frequency | WT n=12 cells/6 rats  KO n=13 cells/6 rats | GLMM | Amplitude p=0.28,  Frequency p=0.61 |
| 5F | vPAG Action potential number | WT n=24 cells/9 rats  KO n=28 cells/10 rats | Two-way repeated measure ANOVA | p=0.54,  F_(1, 17)_=0.38 |
| 5G | vPAG Rheobase potential | WT n=24 cells/9 rats  KO n=28 cells/10 rats | GLMM | p=0.4 |
| 5H | vPAG mEPSC amplitude and frequency | WT n=11 cells/5 rats  KO n=12 cells/6 rats | GLMM | Amplitude p=0.78,  Frequency p=0.88 |
| 6C | Auditory fear conditioning (Tone habituation) | WT=7  KO=8 | Two-way repeated measure ANOVA | p=0.13,  F_(1, 13)_=2.63 |
| 6D | Auditory fear conditioning (Conditioning) |  |  | p=0.54,  F_(1, 13)_=0.74 |
| 6E | Auditory fear conditioning (Recall and extinction) |  | Two-way ANOVA | F_(1,13)_=17.05, p<0.001 |
| 6F | Auditory fear conditioning (Recall and extinction; reanalysed as immobility of paws) |  | Three-way ANOVA | Scoring F_(1,13)_=27.42, p<0.0001; tone F_(1,12)_=3.78, p<0.0001; genotype F_(1,13)_=11.16, p=0.005; scoring x genotype F_(1,13)_=8.03, p=0.014; tone x genotype F_(12,156)_=1.61, p=0.18; scoring x tone F_(12,156)_=1.21, p=0.27; scoring x tone x genotype F_(12,156)_=0.75, p=0.7  **Post hoc two-way ANOVAs:**  WT_(classic)_ vs WT_(paw immobility)_  Scoring F_(1,6)_=3.38, p=0.11; tone F_(12,72)_=1.31, p=0.23; scoring x tone F_(12,72)_=0.94, p=0.51  KO_(classic)_ vs WT_(paw immobility)_  Scoring F_(1,7)_=29.75, p<0.0001; tone F_(12,84)_=3.87, p<0.0001; scoring x tone F_(12,84)_=0.97, p=0.48  WT _(Classic)_ vs KO _(classic)_  Tone F_(12,156)_=1.96, p<0.032; tone x genotype F_(12,156)_=1.48, p=0.13; genotype F_(1,13)_=17.05, p<0.001  WT _(paw immobility)_ vs KO _(paw immobility)_  Tone F_(12,156)_=3.26, p<0.0001; tone x genotype F_(12,156)_=1.07, p=0.39; genotype F_(1,13)_=1.84, p=0.19 |
| 6G | z-scored ERP (during fear recall) |  | Two-way repeated measure ANOVA | p=0.42,  F _(1, 13)_ = 0.73 |
| 6H | z-scored ERP (during tone habituation) |  | Paired t-test | WT: p=0.25  KO: p=0.093 |
| 6 I-L | z-scored ERP (Average of three tones during recall) |  | Paired t-test | Tone 1-3 avg.: WT p=0.0032; KO p=0.0099  Tone 4-6 avg.: WT p=0.003; KO p=0.008  Tone 7-9 avg.: WT p=0.029; KO p=0.004  Tone 10-12 avg.: WT p=0.0158; KO p=0.0046 |
| 7C | Percentage rat escaped from arena | WT=5  KO=9 | Fischer exact test | p<0.0001 |
| 7D | Percentage rat jumping |  |  | p=0.0065 |
| 7E | Classic freezing behaviour |  | Two-way repeated measure ANOVA | p=0.025,  F_(1, 12)_=6.58 |
| 7H | Number of USV calls |  | Mann-whitney test | U=7  p=0.026 |
| 7I | PAG stimulation induced USV calls during freezing |  | Mann-whitney test | U=8  p=0.034 |

**Supplementary table 2. Statistics of supplementary figures.**

| **Figure** | **Task/Measure** | **Population size** | **Statistical test** | **Results/Comparison** |
| --- | --- | --- | --- | --- |
| S1B | Contextual fear conditioning (conditioning) | WT=13  KO=14 | Two-way repeated measure ANOVA | p=0.025,  F _(1, 25)_ = 5.67 |
| S1C | Contextual fear conditioning (recall) |  | Two-way repeated measure ANOVA | p<0.0001,  F _(1, 25)_ = 26.61 |
| S1D | Contextual fear conditioning (recall analysed as immobility of paws) |  | Three-way ANOVA | Scoring F_(1,25)_=200.82, p<0.0001; scoring x genotype F_(1,25)_=0.52, p=.822; time F_(3,75)_=2.68, p=0.53; time x genotype F_(3,75)_=0.392, p=0.59; scoring x time F_(3,75)_=0.224, p=0.879; scoring x time x genotype F_(3,75)_=2.072, p=0.111; genotype F_(1,25)_=20.64, p<0.0001 |
| S2A | Auditory fear conditioning (Conditioning) | WT=12  KO=12 | Two-way ANOVA | p=0.025,  F_(1, 25)_=5.67 |
| S2B | Auditory fear conditioning (Conditioning) implanted rats for LFP | WT=5  KO=8 | Two-way ANOVA | p=0.948 F_(1,11)_=0.004 |
| S2C | % time freezing response during dPAG stimulation | WT=5  KO=9 | Two- way ANOVA | p=0.008,  F_(1, 12)_=9.86 |
| S3A | Distance travelled in open field arena | WT=12  KO=12 | Two-way repeated measure ANOVA | p=0.29,  F_(1, 22)_=1.19 |
| S3C | Distance travelled during habituation phase of APA task | WT=12  KO=11 | One-way ANOVA | p=0.008,  F_(3, 42)_=4.53  **Tukey’s multiple comparisons:**  Trial 1 WT vs *Nlgn3*^-/y^, p=0.99  Trial 2 WT vs *Nlgn3*^-/y^, p=0.90 |
| S3D | Distance travelled during training session 1 of APA task | WT=12  KO=11 | Two-way ANOVA | p=0.5919,  F_(1, 21)_=0.2964 |
| S3E | Marble interaction time | WT=12  KO=10 | Unpaired t-test | p=0.09 |
| S4A | Number of jumps to foot shock | WT=11  KO=14 | Paired t-test | WT: p=0.35  KO: p=0.1 |
| S4B | Tail flick latency | WT=12  KO=10 | Unpaired t-test | p=0.061 |
| S5A | dPAG Resting membrane potential | WT n=25 cells/10 rats  KO n=26 cells/9 rats | GLMM | p=0.61 |
| S5A | vPAG Resting membrane potential | WT n=24 cells/10 rats  KO n=28 cells/9 rats | GLMM | p=0.75 |
| S5B | dPAG input resistance | WT n=25 cells/10 rats  KO n=26 cells/9 rats | GLMM | p=0.09 |
| S5B | vPAG input resistance | WT n=24 cells/9 rats  KO n=28 cells/10 rats | GLMM | p=0.26 |
| S5C | dPAG membrane time constant | WT n=25 cells/10 rats  KO n=26 cells/9 rats | GLMM | p=0.78 |
| S5C | vPAG membrane time constant | WT n=24 cells/9 rats  KO n=28 cells/10 rats | GLMM | p=0.0095 |
| S5D | dPAG capacitance | WT n=25 cells/10 rats  KO n=26 cells/9 rats | GLMM | p=0.11 |
| S5E | dPAG Action potential threshold | WT n=25 cells/10 rats  KO n=26 cells/9 rats | GLMM | p=0.86 |
| S5E | vPAG Action potential threshold | WT n=24 cells/9 rats  KO n=28 cells/10 rats | GLMM | p=0.47 |
| S5F | dPAG depolarisation rate | WT n=25 cells/10 rats  KO n=26 cells/9 rats | GLMM | p=0.71 |
| S5F | vPAG depolarisation rate | WT n=24 cells/9 rats  KO n=28 cells/10 rats | GLMM | p=0.9 |
| S5G | dPAG repolarisation rate | WT n=25 cells/10 rats  KO n=26 cells/9 rats | GLMM | p=0.76 |
| S5G | vPAG repolarisation rate | WT n=24 cells/9 rats  KO n=28 cells/10 rats | GLMM | p=0.9 |
| S5H | dPAG fast afterhyperpolarisation potential | WT n=25 cells/10 rats  KO n=26 cells/9 rats | GLMM | p=0.0047 |
| S5H | vPAG fast afterhyperpolarisation potential | WT n=24 cells/9 rats  KO n=28 cells/10 rats | GLMM | p=0.58 |
| S6A | dPAG action potential number | WT n=15 cells/7 rats  KO n=6 cells/4 rats | Two-way repeated measure ANOVA | p=0.0094,  F_(1, 9)_=10.82 |
| S6B | vPAG action potential number | WT n=14 cells/7 rats  KO n=6 cells/4 rats | Two-way repeated measure ANOVA | p=0.92,  F_(1, 13)_=0.0097 |
| S7A | Average Z-scored peak to trough amplitude | WT n=7  KO n=8 | Pearson’s R test | WT p=0.63, r=-0.22  KO p=0.41, r=-0.34 |
| S7B | Average peak to trough duration |  | Pearson’s R test | WT p=0.61, r=0.23  KO p=0.23, r=0.47 |
| S7D | LFP peak to trough duration |  | Two-way repeated measure ANOVA | p=0.042,  F_(1, 13)_=5.09 |
